# Multi-objective optimization of sustainable cement-zeolite improved sand based on life cycle assessment and artificial intelligence

**DOI:** 10.12688/f1000research.148275.1

**Published:** 2024-04-10

**Authors:** Sepideh Nasrollahpour, Amin Tanhadoust, Satinder Kaur Brar, Hossein MolaAbasi, Moncef L. Nehdi, Omolbanin Ataee

**Affiliations:** 1 Department of Civil Engineering, Lassonde School of Engineering, York University, Toronto, Ontario, Canada; 2 Department of Civil Engineering, Isfahan University of Technology, Isfahan, Iran; 3 Department of Civil Engineering, Gonbad Kavous University, Gonbad Kavous, Golestan, Iran; 4 Department of Civil Engineering, McMaster University, Hamilton, Ontario, Canada; 5 Department of Geography and Urban Planning, University of Mazandaran, Babolsar, Mazandaran, Iran

**Keywords:** Zeolite; Cemented Sand; Life Cycle Assessment; Artificial Neural Network; Multi-Objective Optimization.

## Abstract

**Background:**

Cement-zeolite improved sand can be used in diverse civil engineering applications. However, earlier research has not duly optimized its production process to attain best mechanical strength, lowest cost, and least environmental impact. This study proposes a multi-objective optimization approach using back-propagation neural network (BPNN) to predict the mechanical strength, along with an adaptive geometry estimation-based multi-objective evolutionary algorithm (AGE-MOEA) to identify the best parameters for cement-zeolite-improved sand, filling a long-lasting research gap.

**Methods:**

A collection of unconfined compression tests was used to evaluate cemented sand specimens treated with stabilizers including portland cement (at dosages of 2, 4, 6, 8, and 10%) and six dosages of natural zeolite as partial replacement for cement (0, 10, 30, 50, 70, and 90%) at different curing times of 7, 28, and 90 days. The study further conducts a detailed analysis of life cycle assessment (LCA) to show how partial zeolite replacement for cement impacts the environment. Through a tuning process, the BPNN model found the optimal architecture and accurately predicted the unconfined compressive strength of cement-zeolite improved sand systems. This allowed the AGE-MOEA to optimize zeolite and cement dosages, density, curing time, and environmental impact.

**Results:**

The results of this study reveal that the optimal range of zeolite was between 30-45%, which not only increased cemented sand strength, but also reduced the cost and environmental impact. It is also shown that increasing the zeolite replacement to 25-30% can increase the ultimate strength of cemented sand, yet exceeding this limit can cause the strength to decrease.

**Conclusions:**

Zeolite has the potential to serve as an alternative for cement in applications that involve cemented sand, while still achieving mechanical strength performance, which is comparable or even superior. From an LCA standpoint, using zeolite as partial cement replacement in soil improvement projects is a promising alternative.

## Introduction

**Figure 1.**
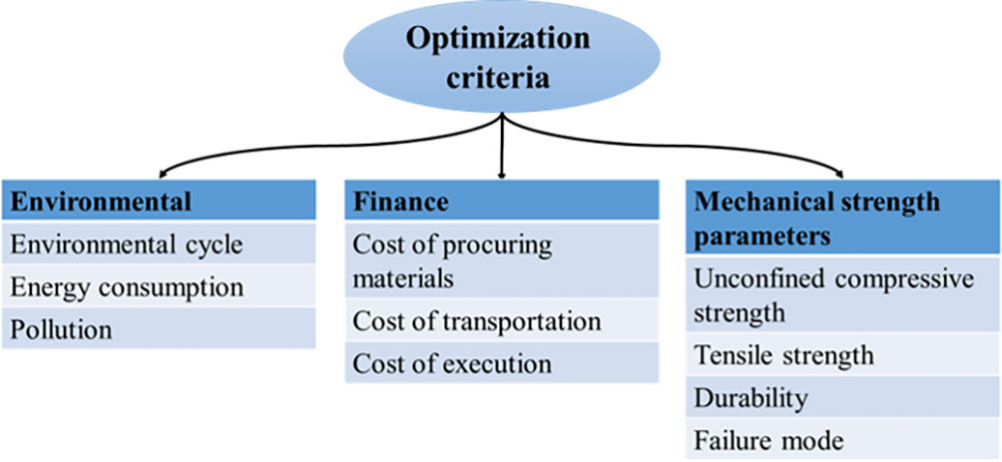
Key criteria for optimal selection of materials and implementation methods.

A paramount and current issue faced by geotechnical engineers in the field of soil stabilization is determining sustainable practices and making best use of the materials during the implementation of multiple solutions considered earthworks, retaining walls, construction of dams and roads, and other projects. ^1^ Considerations for pertinent optimization approaches are depicted in Figure 1. It can be observed in this figure that several criteria influence the selection of materials and implementation methods. In general, mechanical, financial, and environmental indices are among the most important criteria for such projects. The influence of various materials on the mentioned criteria is diverse. For example, using cement stabilizers in soil improvement projects can make the soil stronger, yet may not meet other criteria, especially those that pertain to environmental impacts. ^2^

Pertinent studies on soil improvement methods and the use of additives in the open literature primarily deal with strength criteria, define a series of mixing schemes, and then examine mechanical soil improvement parameters. The parameters defined in the mixing plan are usually the dosage of the additive, density, time, and curing conditions. The optimization of additive values and density for specific curing times in such projects is often accomplished by providing a key relationship based on laboratory results, and then, based on the minimum strength value to provide bearing capacity or stability as per the defined optimal parameters provided. ^3^

For instance, Consoli *et al*. ^4^ presented the key parameter of η/C (in which η and C are the porosity and cement percent, respectively) to determine the strength parameters as $\mathrm{UCS}=a{\left(\eta /C\right)}^{b}$ ( *UCS* is unconfined compressive strength, and *a* and *b* are constant values). This relationship is generalized for a wide range of mechanical parameters of other cemented soils, including tensile strength ( *q _t_*), ^5^ accumulative loss of mass ( *ALM*) in wet and dry or freeze and thaw cycles, ^6^^–^^10^ and swelling properties. ^11^^,^^12^ Based on the considered minimum unconfined strength for the base layer near 1000 *kPa*, the value of η/C=1000/a1b is obtained, based on which it is possible to calculate the values of η and *C.* In this regard, MolaAbasi *et al.* ^13^ acknowledged that the addition of 4% cement to the base soil at the same in-situ relative density and for 42 days of curing provides relative strength for the base. Khajeh *et al.* ^14^ used the California bearing ratio ( *CBR*) = 50% as a criterion and presented the related values of density and required materials (cement and zeolite) as *3D* graphs with a similar process for pavements.

The financial criterion, namely the cost of a project, is very important. For instance, in a soil improvement project in a South Africa nuclear site, mixing of the soil with cement and compacting it to a depth of 5.5 meters was more economical than other methods such as deep foundations and replacing the soil with rubble. ^15^ It is worth noting that in the method of replacing local soil with a desirable one, the costs of excavation and moving a large volume of soil are considerable, making this alternative solution less desirable in most cases. ^16^^,^^17^

Over the last two decades, environmental standards have become both more important and more stringent due to industrialization, urbanization, and excessive emissions of greenhouse gases. Environmental considerations include energy consumption, pollution, climate change, conservation of natural resources, depletion of the ozone layer, and environmental cycle assessments of final products. ^18^ For the construction sector in particular, the production of portland cement is carbon intensive. Cement production is responsible for 5 to 8% of the global carbon dioxide ( *CO _2_*) emitted into the atmosphere, which is a crucial factor in climate change. If considered as a country, this industry ranks third largest carbon emitter next to China and the Unites States. It is also the largest user of natural resources, leading to the depletion of aggregates and water resources.

Therefore, partial substitution of cement with pozzolanic materials is an attractive and economical solution deployed in recent years to reduce cement consumption. Pozzolanic materials typically enhance the strength and durability of cement-based materials given proper mixture design and curing. ^19^ Pozzolans typically contain large proportions of *SiO _2_* and possibly *Al _2_O _3_*, which react with the *Ca (OH) _2_* produced in cement hydration to form cementitious products including calcium silicate hydrates ( *C-S-H*) and calcium aluminate hydrates ( *C-A-H*). The advantage of using pozzolans as partial replacement for cement include: i) lower cost, reduced energy consumption reduced pollutants and greenhouse gas emission, increased mechanical strength over time, and enhanced durability.

The most effective pozzolanic materials commonly used in soil remediation practices include fly ash, ^20^ perlite, ^21^ metakaolin, ^22^ silica fume, ^23^ rice husk ash, ^24^ basic oxygen steel slag ( *BOS*) ^25^ and natural zeolite. ^26^ Amongst these, zeolite is one of the most effective pozzolans with large amounts of silica and alumina (more than 60%). It is also a very good absorbent for environmental pollutants owing to its ion exchange capacity and its highly porous structure which has proven to be very effective in heavy metal removal studies. ^27^ Moreover, it reduces the hydraulic conductivity ^28^ and stabilizes and solidifies contaminants. ^29^

Along with durability, serviceability, and mechanical factors, the environmental footprint of engineering products, systems and processes must be properly considered. One helpful technique for determining how systems affect the environment is the life cycle assessment (LCA) approach. ^30^ Throughout a product’s or process’s life cycle, LCA takes inventory of all the resources used and byproducts released into the environment, calculates the cumulative impact on human health and the natural environment, and provides context for those findings through an analysis of the life cycle’s interpretation. From the beginning of production to the latter of its life cycle, ordinary cement has an impact on the environment (cradle-to-grave). As a result, there have been ongoing initiatives to improve its long-term sustainability.

For instance, Huntzinger and Eatmon ^31^ used an LCA software to assess the environmental impacts of four alternative cement manufacturing techniques. They claimed that supplementary cementitious materials (SCM) blended cement had the least negative effects on the environment. ^31^ Alternate materials can be utilized in cement manufacturing to enhance its sustainability. ^32^^–^^35^ Cost reductions may be achieved by selecting proper mixture designs and materials. According to Black, ^36^ however, the most advantageous strategies in terms of CO _2_ eq per unit strength are not always the cheapest. Although cost is a major factor in cement manufacturing, recent improvements in alternate SCMs have enhanced long-term alternatives for developing cost-effective structural systems. ^36^

Some projects have been assessed via environmental standards, including soil improvement methods, ^37^ materials in soil improvement projects, ^38^ and transportation projects. ^2^ In some other studies, a combination of strength, financial, and environmental criteria for sustainable development was considered. For example, Gravina *et al*. ^39^ concluded that 3% lime and a density of 17.44 kN/m ^3^ were optimal models in dispersive soil stabilization using a decision-making model with strength, financial and environmental criteria.

Soil improvement techniques using cement and other additives are often considered for problematic sandy soils, while lime and other additives are more used for fine-grained soils. ^40^ Loose and saturated sands, also found in coastal areas, experience volumetric instability problems and large deformations especially during seismic loads and earthquakes, causing liquefaction. ^41^ Liquefaction is internal instability in which the effective stress decreases as the pore water pressure increases, and the soil becomes fluid during an earthquake, while its shear strength decreases. For instance, the southern shores of the Caspian Sea in Iran’s Mazandaran province have a high liquefaction potential according to the seismic hazards zonation. ^42^ On the other hand, due to the touristic nature of the coastal areas, special attention has been paid to the development of these lands characterized by problematic soils. Hence, soil improvement techniques via additives have been proposed in the form of micro-piles, injection, and deep mixing designs. As mentioned earlier, the use of cement causes numerous environmental problems. Thus, in this study, zeolite was used as partial replacement for cement. Based on the studies of MolaAbasi *et al*., ^2^ Kordnaej *et al*. ^26^ and Jafar Pour *et al*., ^43^ as reported in Table 1, the positive and negative effects of cement, zeolite and density on the three mentioned criteria (strength, cost and environment) are presented.

**Table 1.** Effects of cement, zeolite and density on soil improvement performance indicators.

Independent variable increments	Criteria			
	Cost	Mechanical strength		Environmental
Cement	−	+		-
Zeolite substitution for cement	+	Short term	-	+
		Long term up to 50%	+	
		More than 50%	-	
Density	-	+		-

As shown in Table 1, increasing the cement content increases the cost of the project. For the density parameter, more machinery is needed to work on projects to increase the density, and since most of the machines are diesel-powered, this imposes additional costs and increases environmental pollution. Zeolite, on the other hand, is an environmentally friendly material that allows reducing the proportion of cement and the associated environmental footprint. It is also less costly than cement. However, zeolite is a pozzolanic material and the process of increasing mechanical strength requires additional curing time and using optimal proportions (between 10 and 40% according to previous studies). The main question in this article is which combination of cement, zeolite, and compaction and which curing time would be optimal in terms of higher mechanical strength, lower cost, and reduced environmental impacts. Solving such a multi-objective problems is associated with complexities in input parameters and measurement indices, and is difficult because it is not conducive to simplified analytical solutions and predictors. Accordingly, more sophisticated optimization methodologies are required.

The novelty of this study lies in the combination of life cycle assessment, predictive BPNN, and multi-objective optimization to determine the optimal cement, zeolite, density, and curing time for cement-zeolite improved sand. Prior research on this topic has primarily focused on investigating particular aspects, such as mechanical strength, cost, and environmental effects. This study, however, takes a holistic approach by simultaneously analyzing all three aspects and optimizing them using a multi-objective evolutionary algorithm. In addition, utilizing a BPNN model to predict the unconfined compressive strength of cemented sand adds sophistication to the optimization procedure. This study aims to develop a comprehensive and efficient method for optimizing the properties of cement-zeolite enhanced sand. This study will provide valuable insights into the optimal dosages of cement and zeolite, as well as the optimal curing time and density, by simultaneously considering the mechanical strength, cost, and environmental impacts. In addition, the utilization of a predictive BPNN model and a multi-objective evolutionary algorithm will result in a robust and effective optimization procedure. The manuscript unfolds initially with the delineation of materials and methods, encapsulating a meticulous exposition of the mixture design, life cycle assessment, predictive model formulation, and the optimization procedure. This is succeeded by the results and discussions, elucidating the empirical findings concerning the strength and stiffness attributes, environmental assessment metrics, sensitivity analysis predicated on the predictive model, and the discernment of optimal design alongside decision-making paradigms. The narrative culminates with conclusions, encapsulating the quintessence of the insights gleaned from this inquiry, highlighting the cogent implications for future research on cement-zeolite improved sand and to inform geotechnical and geo-environmental soil improvement engineering applications.

## Materials and methods

### Mixture design

**Figure 2.**
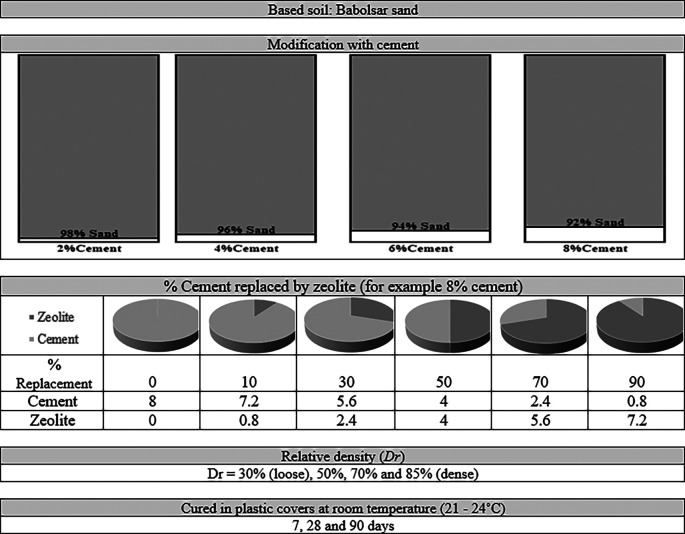
Independent variables used and the process of evaluating resistance criteria.

The soil studied herein is the Babolsar soil, which, according to the unified soil classification system ( *USCS*), is a poorly graded sand ( *SP*). According to geotechnical studies conducted at the Babolsar site, the variation range of relative density is 40 to 55 at low depths of up to 10 meters. Other characteristics of the sandy soil are *G _S_* = 2.72, 0% clay (diameter < 0.002 mm), 5% silt (0.002 < diameter < 0.075 mm), 95% fine sand (0.075 < diameter < 4.72 mm), D _50_ =0.24 mm, $\gamma_{d\;\min}$ = 15.5 kN/m ^3^ and $\gamma_{d\;\max}$ = 17.2 kN/m ^3^. The levels of the independent parameters considered are shown in Figure 2. As shown in this figure, the fabrication process of the samples is presented based on the considered parameters to obtain and evaluate the mechanical strength criteria. Prior work ^29^^,^^44^^,^^45^ has been used to examine the strength and process of calculating the strength criteria. It should also be noted that a study of financial and environmental criteria related to this research has not been performed, which will be described in subsequent sections.

### Life cycle assessment (environmental assessment)

In this section, the approach known as Life Cycle Assessment (LCA) is utilized to determine the impacts that the production of various zeolite-cemented sand mixtures has on the surrounding environment at various stages of the service or product life cycle, including raw materials extraction, production of ingredients, and the transportation of those ingredients to the production site. For this purpose, SimaPro’s database (Ecoinvent 3) was used throughout the investigation. This database contains over 10,000 public processes from the agricultural, energy, detergents, construction, transportation, cardboard, chemical, and waste management sectors. ^46^ In the manufacturing process, production entails every step from raw material extraction to transport to the batch plant, component fabrication, and processing. The SimaPro data set was used to select materials and procedures based on the availability of raw materials as well as expert views. In this study, for assessing and quantifying environmental impacts, different parameters including the number of raw materials for making different cement-sand mixture designs (water, cement, zeolite and sand) taking into account the effect of different relative density percentages and curing time, the transportation distance of raw materials (cement and zeolite) and their packing, and consuming machines (grader, loader, goat roller, sprayer and sprinkler) were considered.

For the LCA method of manufacturing concrete, 16-to-32-ton trucks with EURO3 fuel standards were used to examine the environmental impacts of the transport stage. Cement and zeolite were transported across distances of 76 and 98 kilometers, respectively. The CO _2_ emission factor was calculated as 0.0033-kilogram CO _2_eq per m ^3^ of concrete during the manufacturing and transportation stages. Additionally, 0.009 kg/m ^3^ was assigned to the element that includes temporary structural support and access throughout the manufacturing phase, concrete pumping and installation, and curing. ^30^^,^^47^ The LCA technique was based on ReCiPe 2016. Even though its development was based on CML 2001 and Ecoindicator 99, the ReCiPe approach to life cycle assessment is relatively new. It has three endpoint category indicators that come with normalization factors, and they all measure the damage done to particular protective zones. ^48^ ReCiPe is made up of two groups of impact categories, each with its own set of characterization elements. There are 22 impact categories at the midpoint level, and the majority of these are multiplied by damage factors to form three endpoints.

ReCiPe uses endpoint characterization elements for resources, human health, and ecosystems. It is noted that both the number of years spent being disabled and the number of life years lost are used to determine how healthy an individual is. Ecosystem loss is defined as the loss of species over time and space. These are combined as Disability Adjusted Life Years (DALYs), a metric used by the World Bank and the World Health Organization. Similarly, the term “resources” is defined as the extra costs associated with resource output over an indefinite time horizon (assuming constant yearly production) at a discount rate of 3%. The unit price is $2000 USD. ^49^ The midpoint impact category indicators and the related characterization factors are computed. To compare all groups simultaneously, the values of the impact category were divided into a reference value during normalization. The normalization numbers in SimaPro are modified per citizen. According to the most recent version of the ReCiPe 2016 approach, the world population was utilized as the default number instead of the EU25 + 3 population in SimaPro. The main goal of normalization is to establish the significance of each product and its associated range of outcomes. The normalization findings link the effect category, such as acidification and global warming, to the point in the product life cycle when that category experiences significant environmental issues. Instead, the main objective of a comparative study is to decide which products have the best environmental repercussions. To address this, the normalized values were multiplied by the weighting factors to estimate the relative importance of each effect. In this system, the three damage categories are weighted by a panel at the endpoint level (or damage category level in ISO terms). Each perspective has its own set of weights. The panel evaluation’s average result is the weighting set. ^49^

### Predictive model and performance

*Back Propagation Neural Network*

There are various forms of Artificial Neural Networks (ANN), among which the Back Propagation Neural Network (BPNN) is widely used in engineering problem-solving. Generally, a BPNN contains an input layer, an output layer, and multiple hidden layers. The BPNN training process consists of two phases, Forward propagation, and backward propagation. The signals from the input are received by the hidden layers in the first step, then they are transmitted to the output layer using an activation function as calculated in the following equation ^50^:

$$x_{j}=f_{\mathrm{act}}\left(\sum_{i=1}^{m}w_{\mathrm{ij}}x_{i}+b_{j}\right)$$ (1)

**Figure 3.**
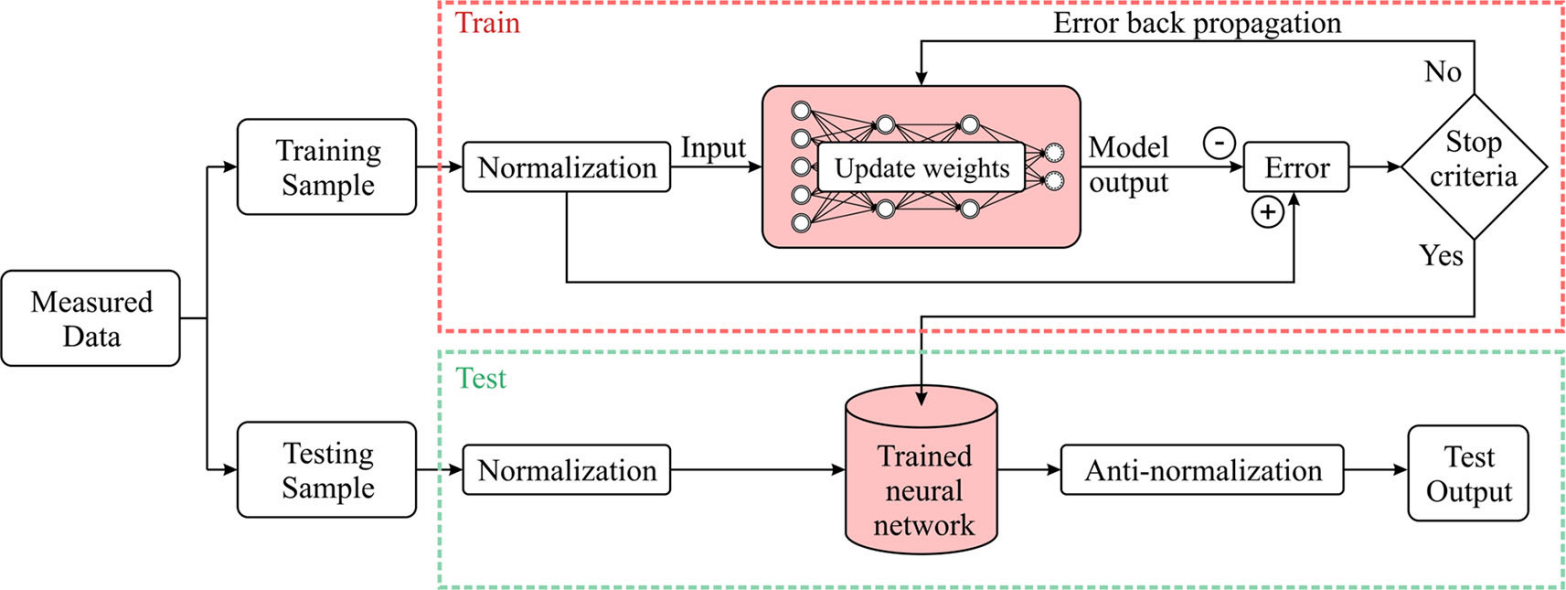
Procedure of back propagation neural network.

Where *m* is the number of neurons, $f_{\mathrm{act}}$ is an activation function, and the output of the *i*th and *j*th neurons are shown as $x_{i}$ and $x_{j}$ respectively; the weight value from neuron *i* to neuron *j* is $w_{\mathrm{ij}}$ and $b_{j}$ is the bias value. ^51^ The second phase, i.e. backpropagation, will be reached if the calculated output does not match the actual output. So, to improve the accuracy of the prediction, the values of weight and bias will be modified to reduce the errors. ^52^ The backpropagation process will stop when the result of the output is less than a specific threshold. The training and prediction process of BPNN is illustrated in Figure 3. ^50^^,^^53^

*Evaluation Metrics*

Three statistical metrics, Mean Squared Error (MSE), Mean Absolute Error (MAE), as well as $R^{2}$ (the coefficient of determination) have been applied to evalutate the accuracy of the model and they are expressed as follows, where, $y_{i}$ denotes the predicted output, $y_{i}^{,}$ is the actual value, and $\bar{y}$ is the mean values for the *n* number of data. ^51^^,^^54^

$$\text{MSE}=\frac{1}{n}\;\sum_{i=1}^{n}{\left(y_{i}^{\prime }-y_{i}\right)}^{2}$$ (2)

$$\text{MAE}=\frac{1}{n}\;\sum_{i=1}^{n}|y_{i}^{\prime }-y_{i}|$$ (3)

$$R^{2}=1-\frac{\sum_{i=1}^{n}{\left(y_{i}^{\prime }-y_{i}\right)}^{2}}{\sum_{i=1}^{n}{\left(y_{i}-\bar{y}\right)}^{2}}$$ (4)

*Proposed model*

**Figure 4.**
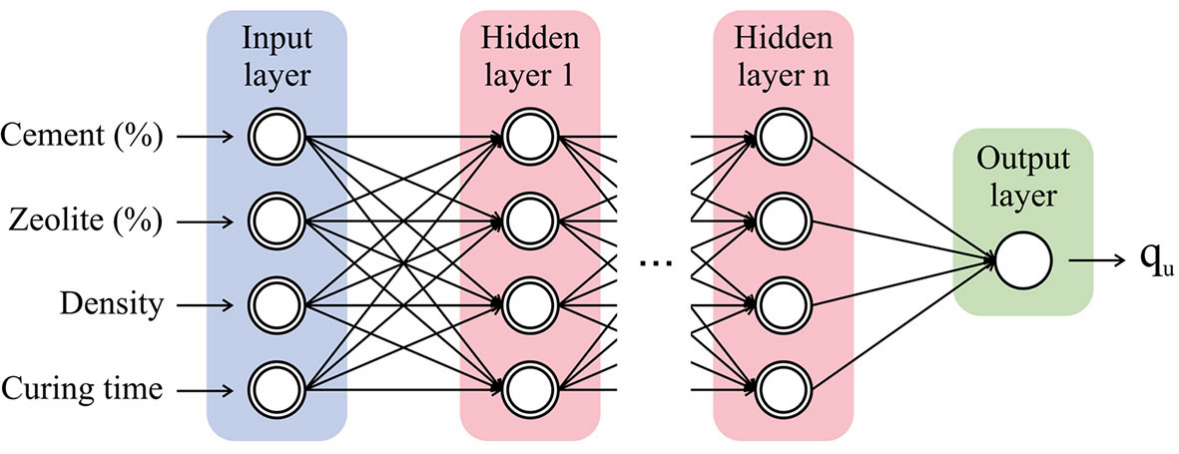
Proposed BPNN predictive model.

Compared to other ANN models, BPNN can achieve great efficiency and computational reliability. ^50^ Several BPNNs were developed in the current work using 27 various architectures to explore the optimal arrangement of neurons and layers, as shown in Table 2. The Rectified Linear Unit (ReLU) was used as the activation function, and 0.001 was chosen as the learning rate. In addition, for all layers and each learning phase, a batch size of 32 was set. A data collection derived from 216 individual experimental observations was used for the training and validation of BPNNs. The input values consist of five key independent parameters, three of which are mixture parameters and the curing time. As shown in Figure 4, the four input variables are cement (%), zeolite (%), density, and curing time (days), whereas the output variable is the soil strength ( *q _u_*).

**Table 2.** Architecture parameters and evaluation metrics of proposed BPNNs.

	Parameters of network architecture			Evaluation metrics		
Architectures	Number of layers	Number of hidden layers	Number of units (in each hidden layer)	MAE mean (std.)	MSE mean (std.)	*R* ^2^ mean (std. ×10 ^−3^)
A-1	4	2	100, 50	20.074 (4.637)	0.686 (0.256)	0.9983 (0.65)
A-2			200, 50	**13.989** ( **2.910**)	**0.411** ( **0.167**)	**0.9990** ( **0.43**)
A-3			200, 100	16.251 (5.290)	0.532 (0.349)	0.9987 (0.84)
A-4			300, 100	14.920 (4.666)	0.524 (0.407)	0.9987 (0.98)
A-5			300, 200	17.025 (9.104)	0.822 (0.956)	0.9980 (2.41)
A-6			300, 300	16.645 (7.556)	0.686 (0.681)	0.9983 (1.62)
A-7			400, 400	14.761 (6.530)	0.515 (0.542)	0.9987 (1.34)
A-8			400, 50	15.413 (5.324)	0.520 (0.330)	0.9987 (0.77)
A-9			400, 200	15.649 (9.002)	0.623 (0.806)	0.9985 (1.84)
B-1	5	3	100, 50, 50	29.805 (9.288)	1.560 (0.725)	0.9961 (1.83)
B-2			200, 100, 50	18.183 (3.393)	0.634 (0.403)	0.9984 (1.03)
B-3			300, 200, 100	17.658 (9.297)	0.950 (0.981)	0.9976 (2.52)
B-4			400, 300, 200	15.071 (6.519)	0.759 (0.803)	0.9981 (2.05)
B-5			300, 300, 300	14.620 (8.136)	0.793 (1.030)	0.9980 (2.65)
B-6			200, 300, 400	14.360 (5.545)	0.599 (0.653)	0.9985 (1.65)
B-7			100, 200, 300	15.250 (3.068)	0.509 (0.190)	0.9987 (0.51)
B-8			50, 100, 200	34.592 (6.845)	1.919 (0.576)	0.9952 (1.49)
B-9			50, 50, 100	37.305 (10.933)	2.274 (1.177)	0.9944 (2.74)
C-1	6	4	100, 100, 50, 50	26.808 (5.396)	1.488 (0.685)	0.9964 (1.57)
C-2			200, 100, 100, 50	19.669 (4.376)	0.766 (0.494)	0.9982 (1.09)
C-3			300, 200, 100, 50	23.410 (7.426)	1.361 (1.165)	0.9966 (3.13)
C-4			400, 300, 200, 100	17.485 (6.942)	0.937 (0.997)	0.9977 (2.63)
C-5			300, 300, 300, 300	17.869 (10.648)	1.289 (2.214)	0.9969 (5.40)
C-6			100, 200, 300, 400	22.038 (8.569)	1.325 (1.178)	0.9966 (3.19)
C-7			50, 100, 200, 300	35.853 (11.896)	2.363 (1.447)	0.9942 (3.82)
C-8			50, 100, 100, 200	32.749 (9.735)	2.009 (1.342)	0.9951 (3.51)
C-9			50, 50, 100, 100	47.715 (6.742)	3.818 (0.992)	0.9908 (2.32)

Table 3 presents the maximum and minimum range of inputs and outputs of the BPNNs. In addition, 162 (75%) and 54 (25%) data points were chosen randomly from the dataset for the training and testing phases, respectively. A normalization approach was employed to ensure that the layers’ inputs are adjusted within a particular range to achieve higher performance in the BPNN training operation. Also, a drop-out probability rate of 10% was added in the neural connection to prevent overfitting during the training stage. Finally, each architecture was trained and evaluated for 10 individual repetitive processes to investigate the stability and reliability of BPNNs.

**Table 3.** Ranges of inputs and outputs of neural network.

	Variable	Unit	Minimum	Maximum
Input	Cement	%	2	8
	Zeolite	%	0	90
	Dr	%	50	85
	Curing time	day(s)	7	56
Output	*q _u_*	kPa	0	2760.8

**Figure 5.**
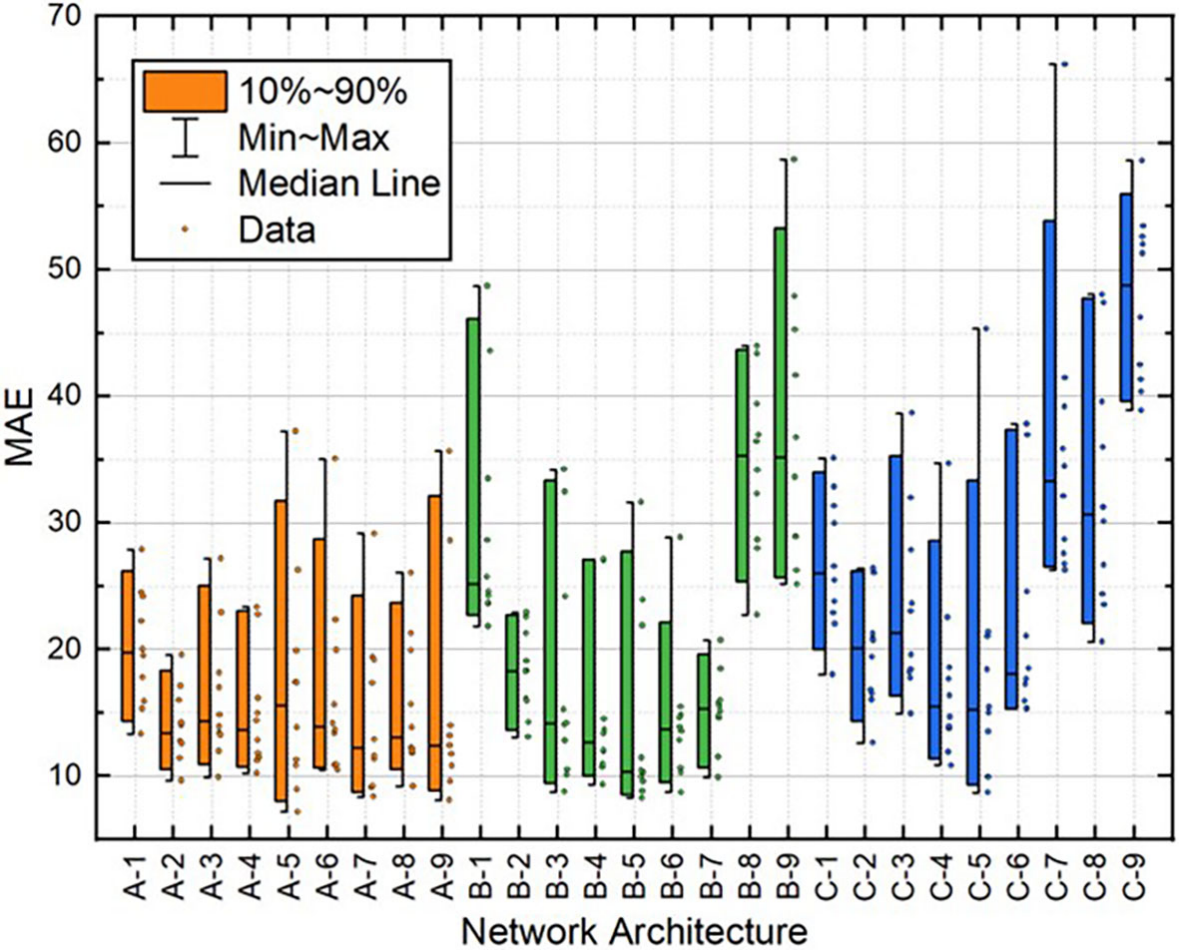
Distribution of MAE values for train dataset.

**Figure 6.**
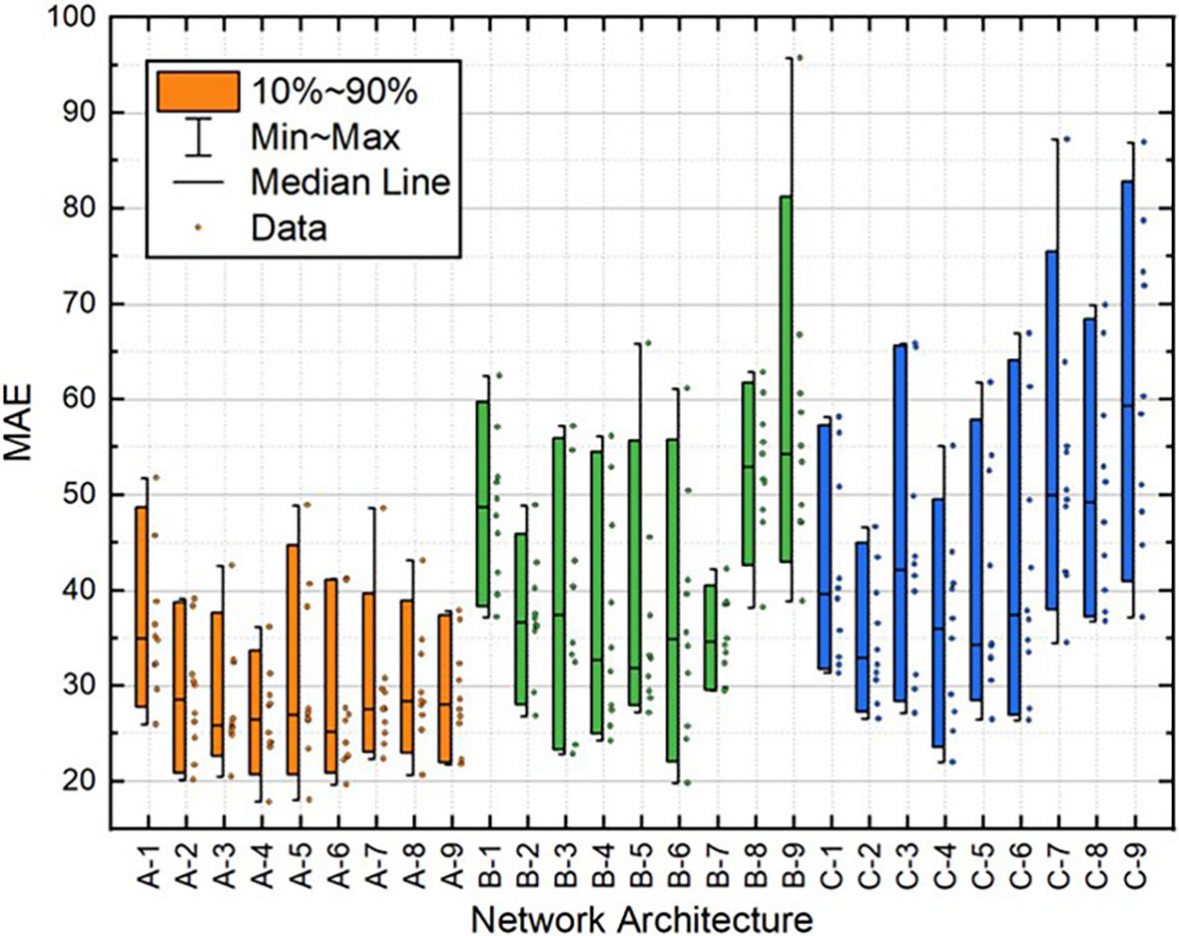
Distribution of MAE values for test dataset.

**Figure 7.**
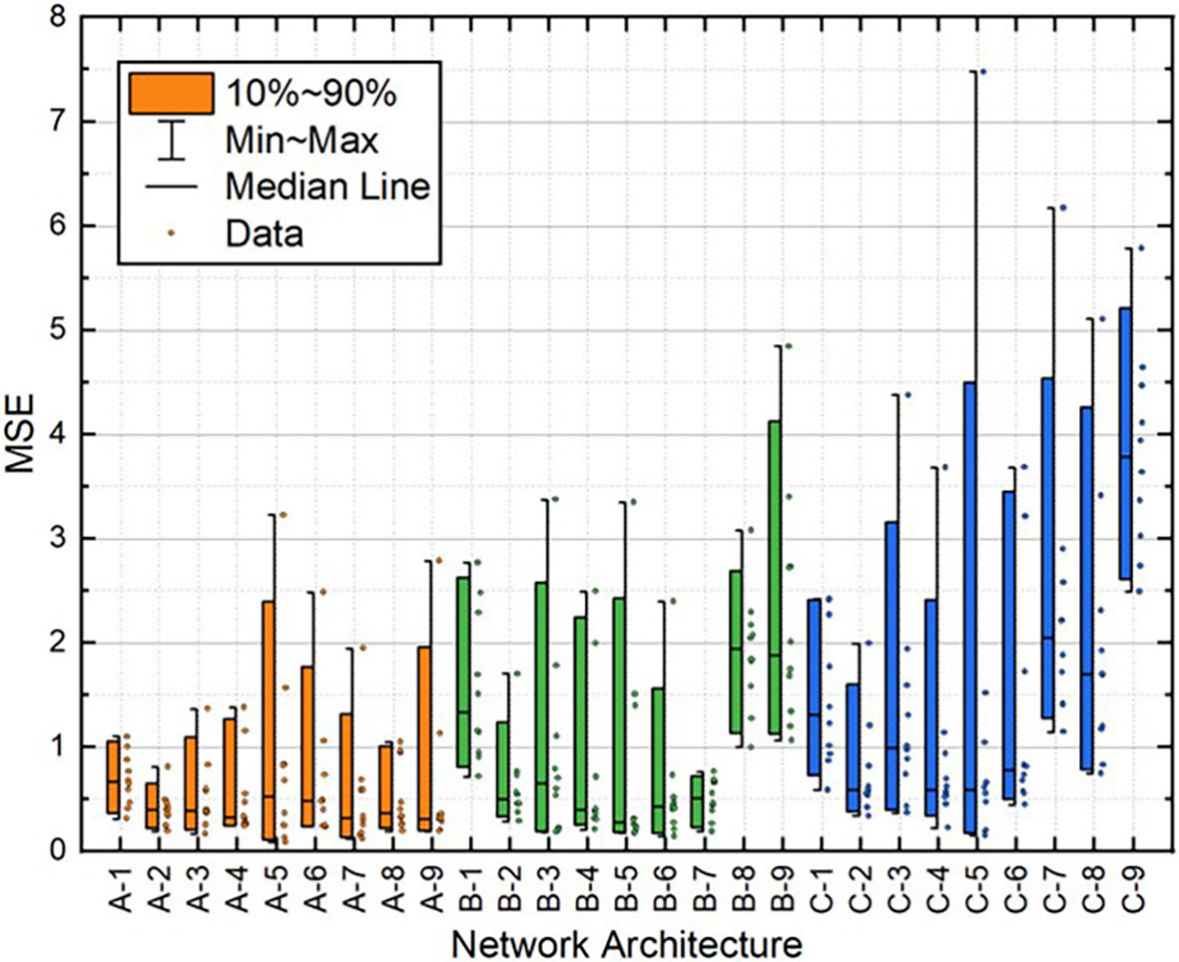
Distribution of MSE values for train dataset.

**Figure 8.**
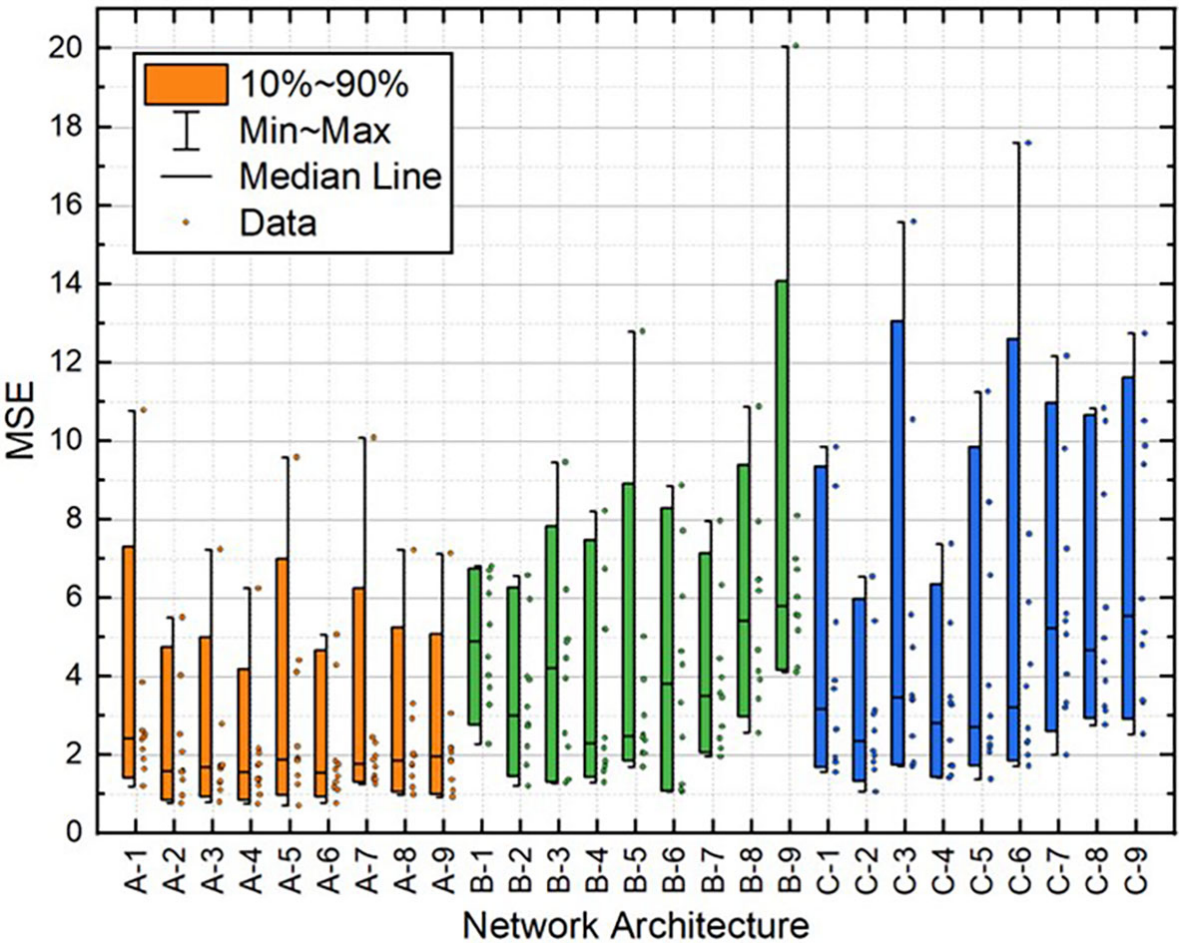
Distribution of MSE values for the test dataset.

**Figure 9.**
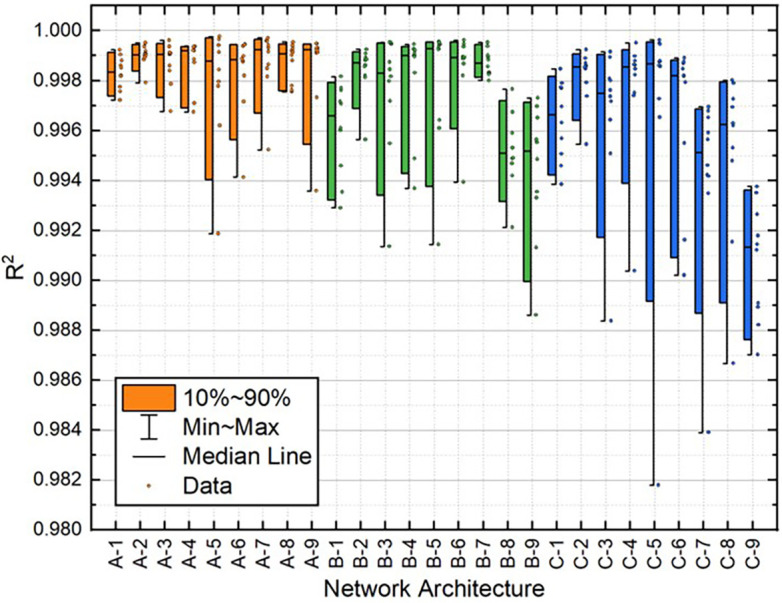
Distribution of *R* ^2^ values for train dataset.

**Figure 10.**
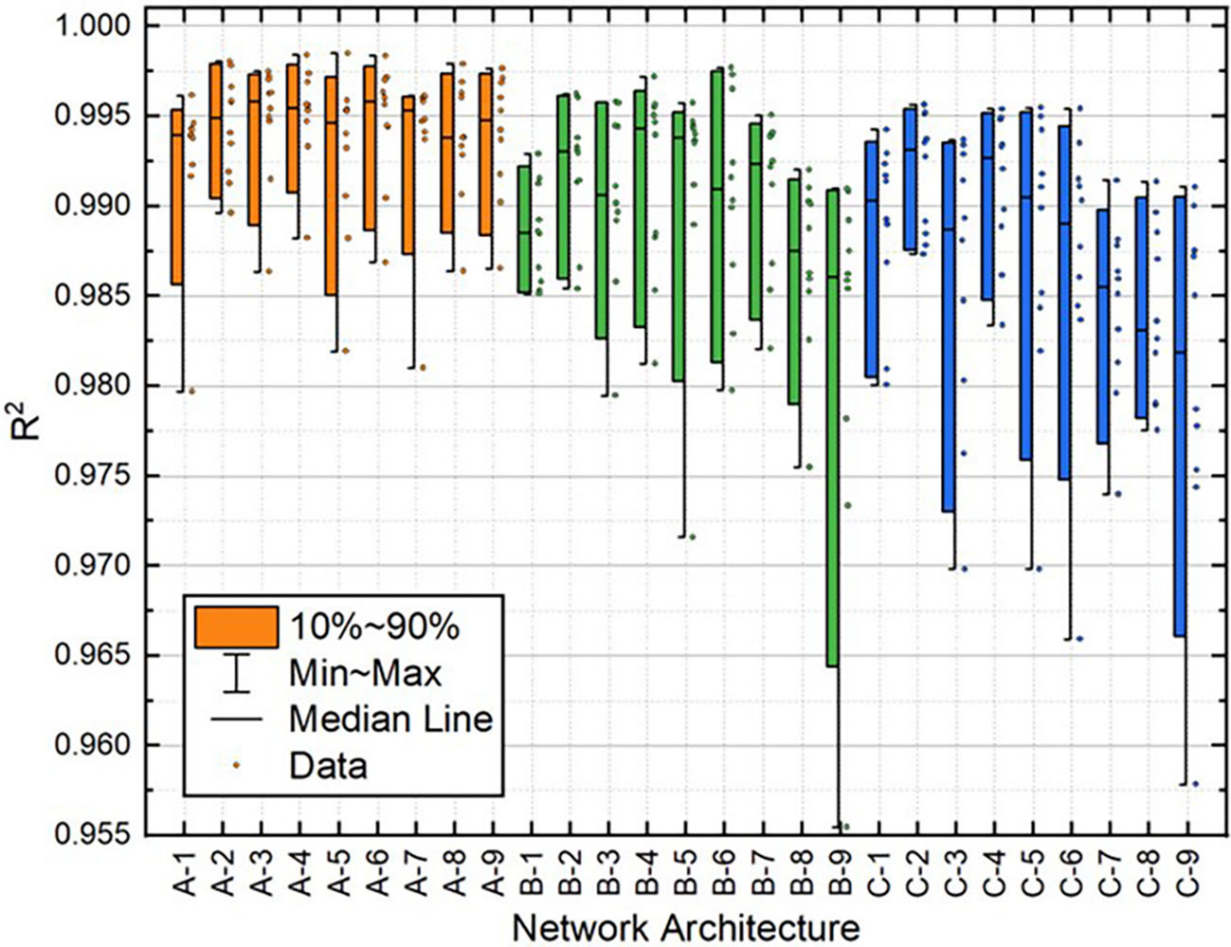
Distribution of *R* ^2^ values for test dataset

**Figure 11.**
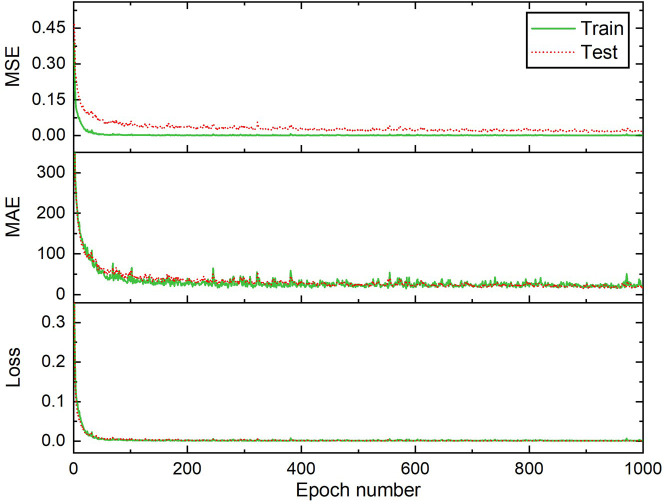
Performance of proposed BPNN predictive model (A-2 architecture).

**Figure 12.**
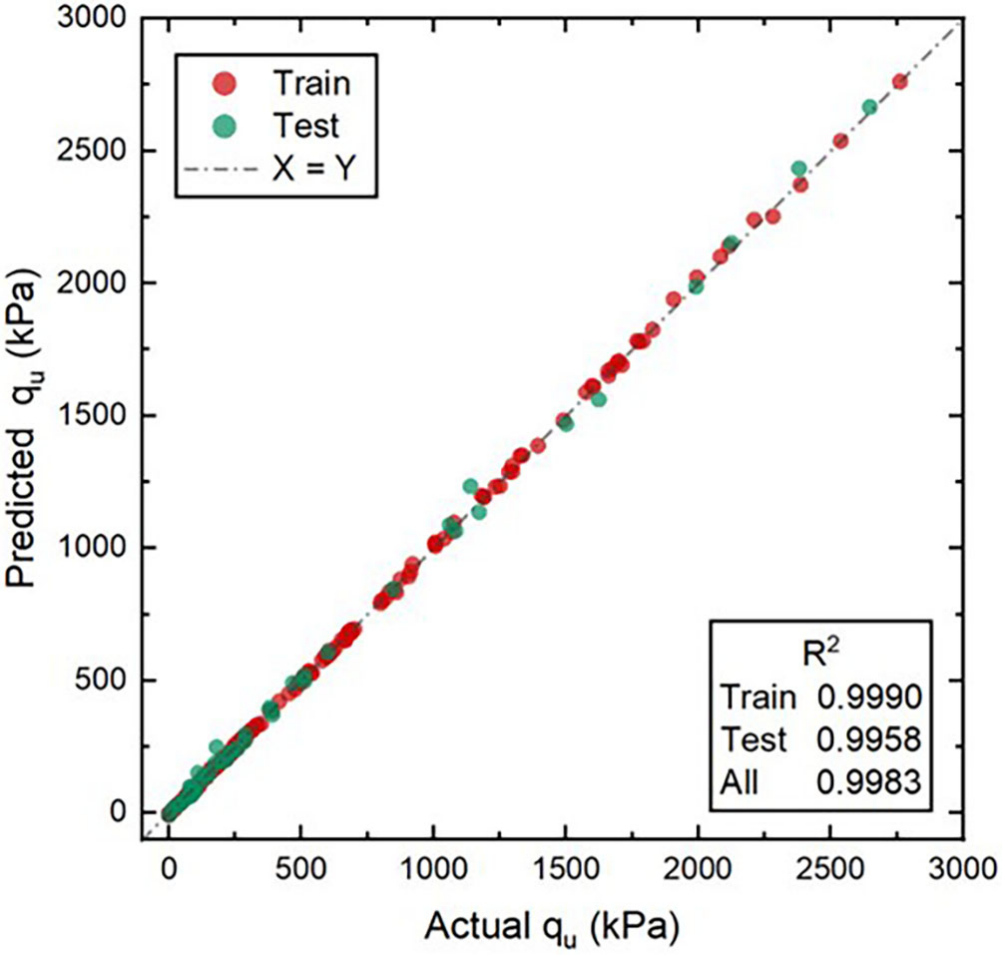
Predicted and actual values of UCS (A-2 architecture).

The average and standard deviation of the *R* ^2^, MAE, and RMSE values for all 27 architectures are reported in Table 2. Moreover, the distribution of these variables for all 10 individual runs for each BPNN is depicted in Figures 5 to 10. These results demonstrate that the A-2 outperformed the other architectures in terms of statistical metrics values and stability. Figure 11 illustrates the values of loss, MAE, and MSE during the progress of BPNN training for the A-2 model. It can be concluded that the network training is steady, and the metrics values for the train and test datasets are quite close, indicating that the network was not over-trained. Despite obtaining a better maximum *R* ^2^, the A-2 model had the best minimum, average, and distribution of *R* ^2^, RMSE, and MAE. Figure 12 depicts the linear regression for the predicted and actual values of soil strength using the model with the best performance. As a result, the proposed BPNN model was shown to accurately predict the ultimate strength of the studied sand.

### Optimization procedure and performance

*Optimization algorithm*

Most engineering problems in the real world have multiple objectives to balance simultaneously, for instance maximizing the performance and reliability along with minimizing the cost and environmental footprint could be a complex problem since these conflicting objectives can prevent simultaneous optimization. Genetic Algorithm (GA), which is a metaheuristic method inspired by evolutionary theory, is often appropriate for this type of multi-objective optimization problems (MOPs). ^55^ The goal of multi-objective evolutionary algorithms (MOEAs) is to approach the optimal Pareto front (PF) with a collection of non-dominated solutions in a single run, so MOEAs make non-dominated fronts, which is not only distributed well over the optimal PF (diversity), but also close to the PF as much as possible (proximity). ^56^ One of the most well-known MOEA based on Pareto dominance is NSGA II (Non-dominated Sorting Genetic Algorithm), whose diversity and proximity is achieved using the crowding distance and the Pareto-dominance, respectively. ^56^ The rapid non-dominated sorting process of NSGA II in its selection is a distinguishing characteristic of its ranking solution. ^57^

Adaptive Geometry Estimation based Multi-Objective Evolutionary Algorithm (AGE-MOEA) is an optimization framework based on NSGA-II yet differs from it in replacing the crowding distance of NSGA-II by a survival score, for which calculations need the diversity and proximity of non-dominated sets. To simplify the computation of the survival score, in each generation, the geometry of the initial non-dominated subset is estimated by AGE-MOEA, afterwards, this estimation which gets more accurate as the algorithm matures, is used as the geometry of the Pareto set. ^56^

*Optimization framework*

The objective functions and optimization constraints that need to be specified in any optimization problem. In the present framework, the UCS, LCA, and cost are the three values of objective functions. Moreover, the boundary values of design variables are considered as constraints. Thus, the MOO problem is defined as follows:

**Table:** 

Minimize:	$F_{\text{Cost}}\left(x\right)$	
	$F_{\mathrm{LCA}}\left(x\right)$	
Maximize:	$q_{u}\left(x\right)$	
Subjected to:	${\mathrm{lb}}_{i}<x_{i}<{\mathrm{lb}}_{i}$	i=1,2,3,4

where the $F_{\text{Cost}}$ , $F_{\mathrm{LCA}}\left(x\right)$ , and $q_{u}\left(x\right)$ are the cost, LCA, and ultimate strength of the cemented sand, respectively. Furthermore, x denotes the vector of four main independent design variables, while ${\mathrm{lb}}_{i}$ and ${\mathrm{ub}}_{i}$ are the minimum and maximum of $x_{i}$ , respectively. The simpler alternative optimization problem is defined accordingly, while the value of the curing time is implemented manually in the range of 7, 14 … 56, to better compare between optimal variables. It is noted that the $q_{u}\left(x\right)$ is predicted by the trained BPNN model with the best performance (A-2). Because the development of an optimized and robust algorithm is an error-prone technique, Pymoo, ^58^ a sophisticated open-source Python framework for multi-objective optimization, is used. A population size of 100 was chosen for the optimization procedure until 200 generations for AGE-MOEA. In addition to the main optimization, 8 further optimizations were performed independently with curing times ranging from 7 to 56 days. Figure 13 depicts the values of the objective functions in the optimization procedure to illustrate the convergence and performance of the proposed optimization framework.

**Figure 13.**
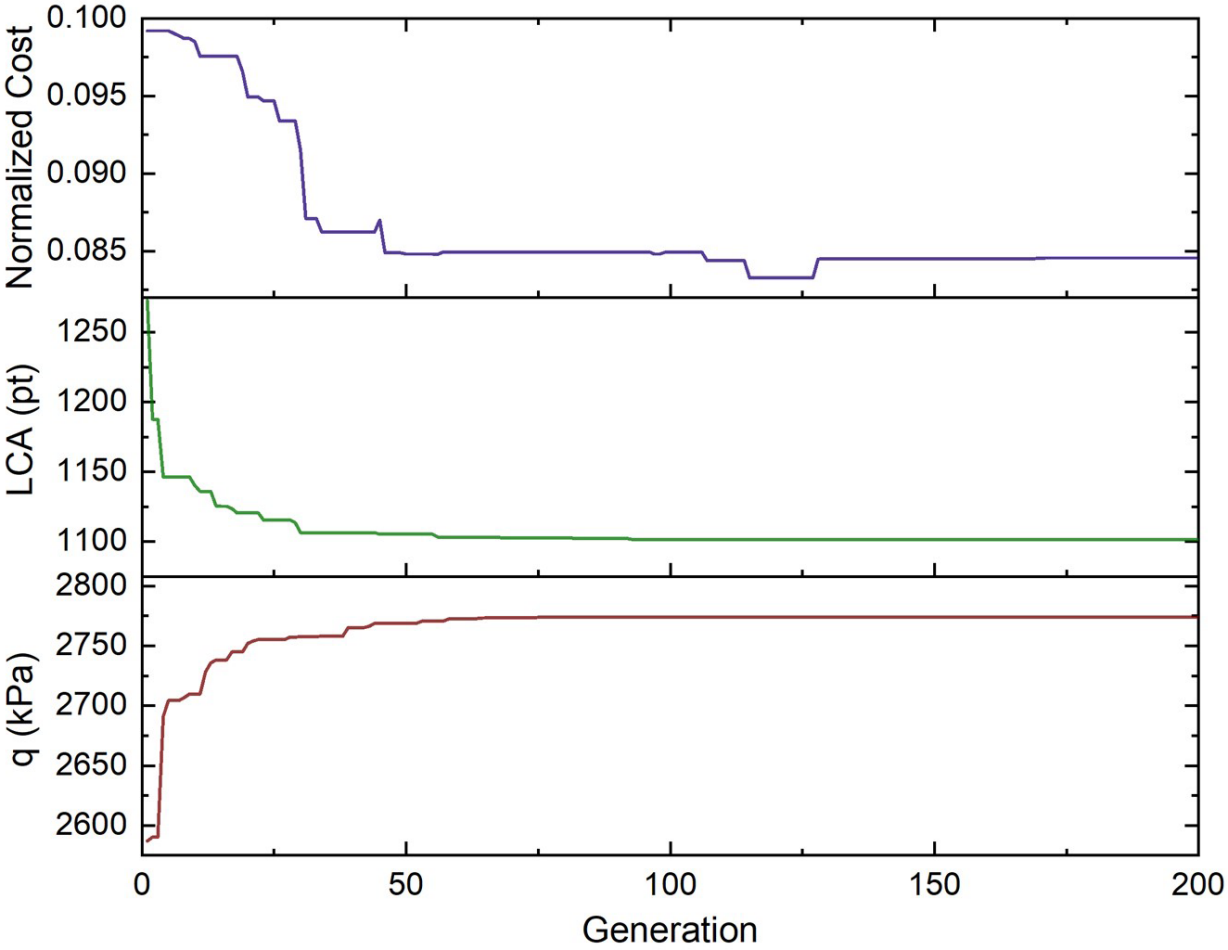
Performance of proposed optimization framework.

## Results

### Strength and stiffness

**Figure 14.**
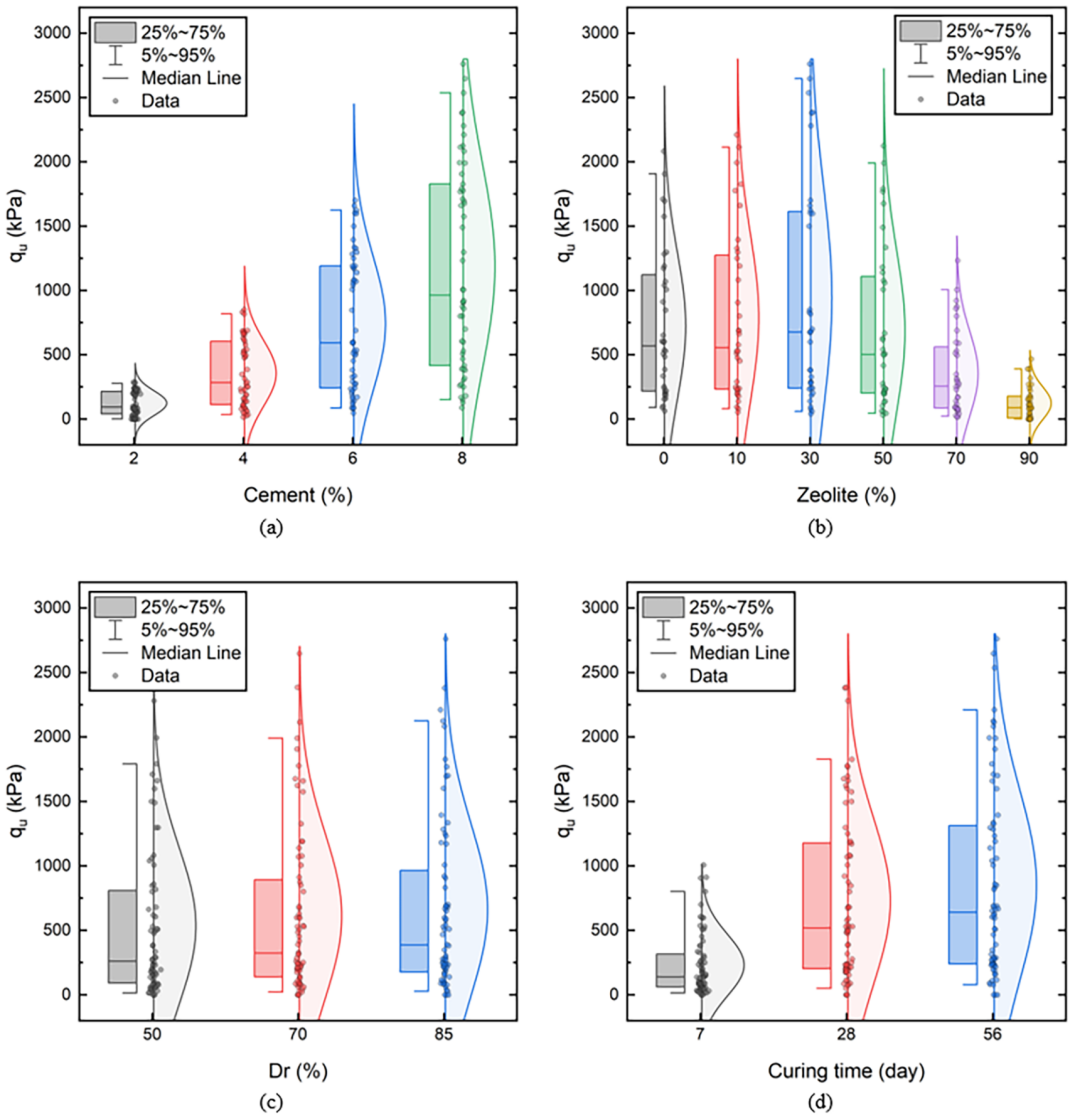
Schematic simple correlation between input and output variable UCS considering variation in: a) Cement, b) Zeolite, c) Dr, and d) Curing time.

Strength parameters including UCS, *q _t_*, and wet/dry cycle durability of sand-cement-zeolite mixtures and the relationships presented by researchers in this field, which consider the effects of each parameter, including cement and zeolite percentage, curing time, and Dr percentage. ^59^ It is worth mentioning that earlier experimental results of the authors ^44^^,^^45^ have also been used to examine the strength criteria and the process of calculating the *UCS.* The descriptions of data distribution are presented in Figure 14.

### Life cycle assessment

**Figure 15.**
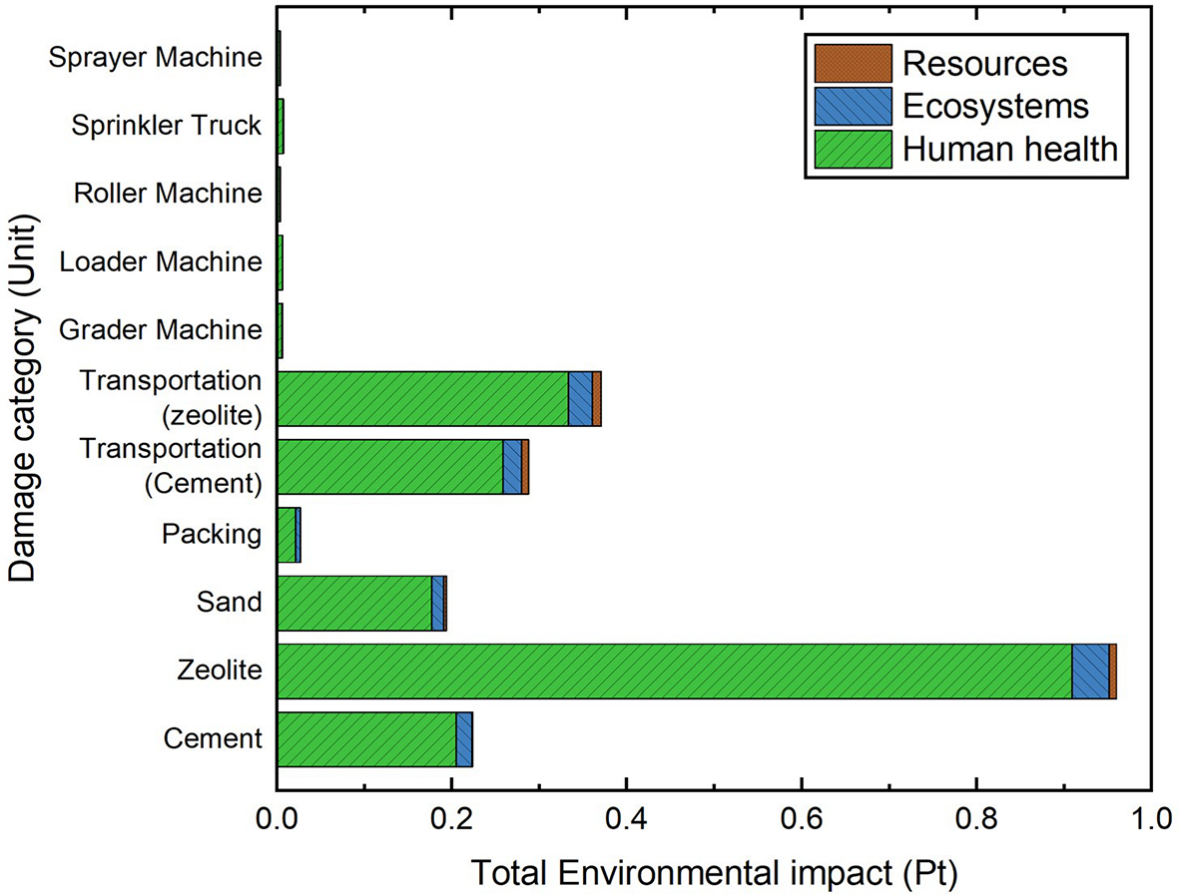
Zeolite cemented sand single score section.

**Figure 16.**
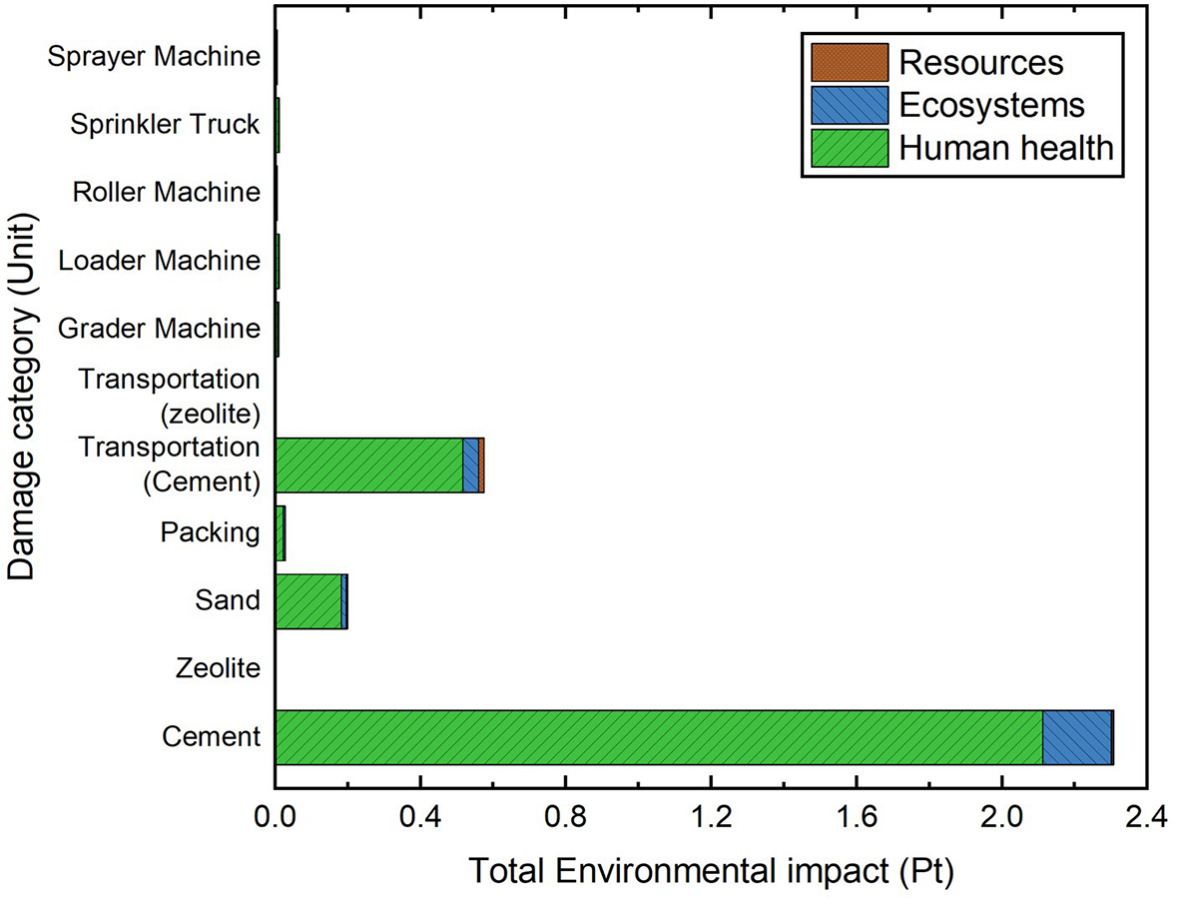
Cemented sand single score section.

Figure 15 and Figure 16 show a comparison of cemented sand and zeolite cemented sand for two mixture designs, which depict the weighted and single score of the output of SimaPro, respectively. In Table 4, a comparison of the LCA result in the production process of one cubic meter of cemented sand and zeolite cemented sand is shown. Zeolite is proven as an environment-friendly material for cement substitution, with less negative impact on ecosystem and human health, and a total ( *Pt*) that decreases to a significant degree. It is recognized that zeolite extraction has a relatively insensible degree of environmental and health damage compared to cement production.

**Table 4.** Comparison of life cycle assessment results at the impacts weighting stage in the process of production of one cubic meter of cemented sand and zeolite cemented sand using the ReCiPe method.

	Production	
Damage category	Cemented sand	Zeolite cemented sand
Human health	2.926	1.922
Ecosystems	0.260	0.132
Resources	0.027	0.030
Total (Pt)	3.214	2.084

### Sensitivity analysis based on predictive model

**Figure 17.**
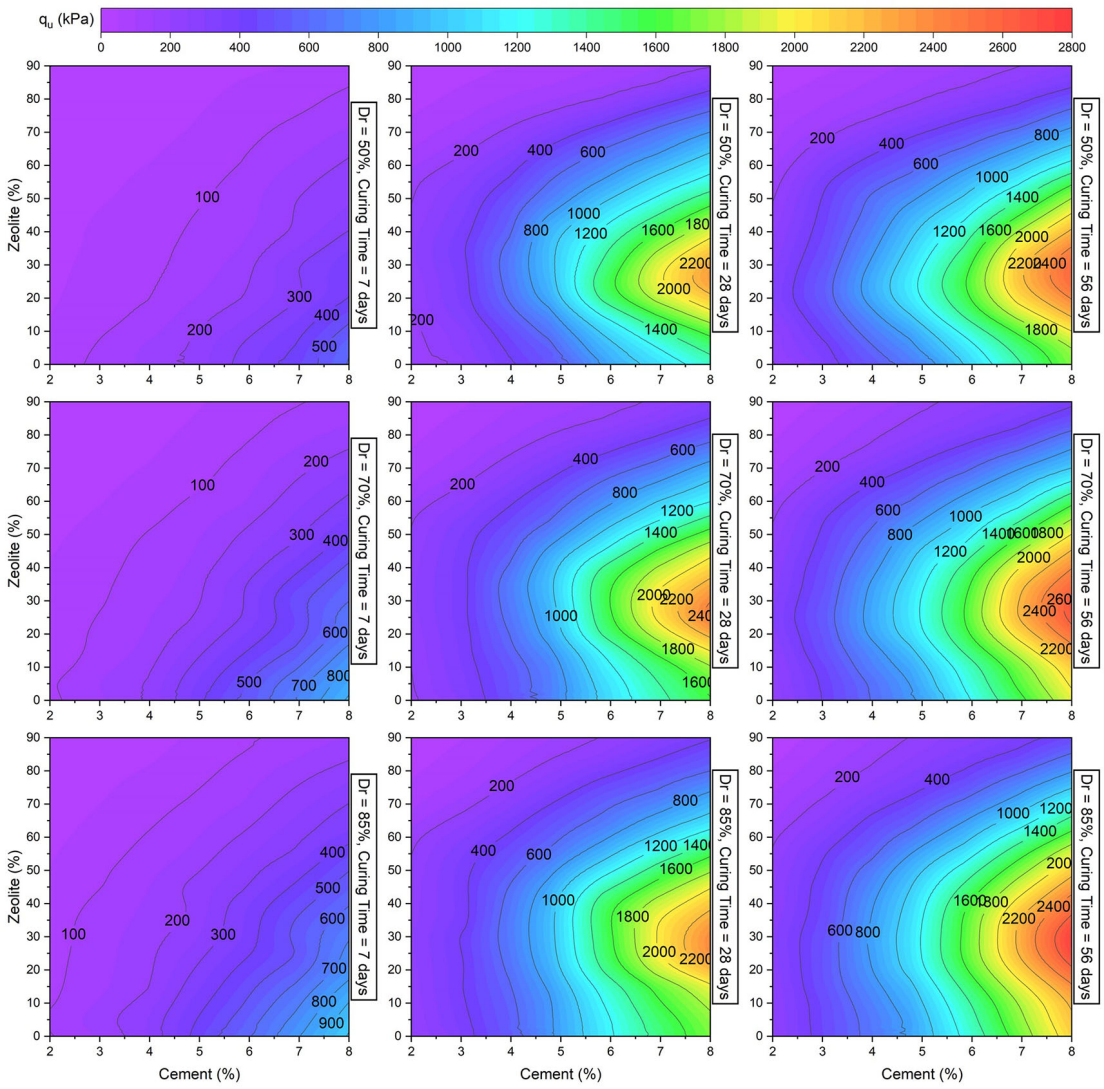
Multivariate contour of UCS versus cement and zeolite.

The model predicted unconfined compressive strength results of cemented sand are plotted in Figure 17 for different values of cement and zeolite replacement and different relative density with various curing times. It can be observed that adding more cement to the sand mixture increased the strength under all conditions. Moreover, higher cement percentage increased the *q _u_* with increased Dr and curing time. Furthermore, by increasing the zeolite replacement at curing times of 28 and 56 days, the strength of the sand increased to a maximum peak value and then decreased. This indicates that there is an optimal value of zeolite replacement ratio for a curing time of more than 7 days. It can also be observed that the optimal range of zeolite replacement ratio for longer curing times is in the 20 to 35% range. It can be further concluded that the *q _u_* tends to increase to some extent by increasing the *Dr.*

**Figure 18.**
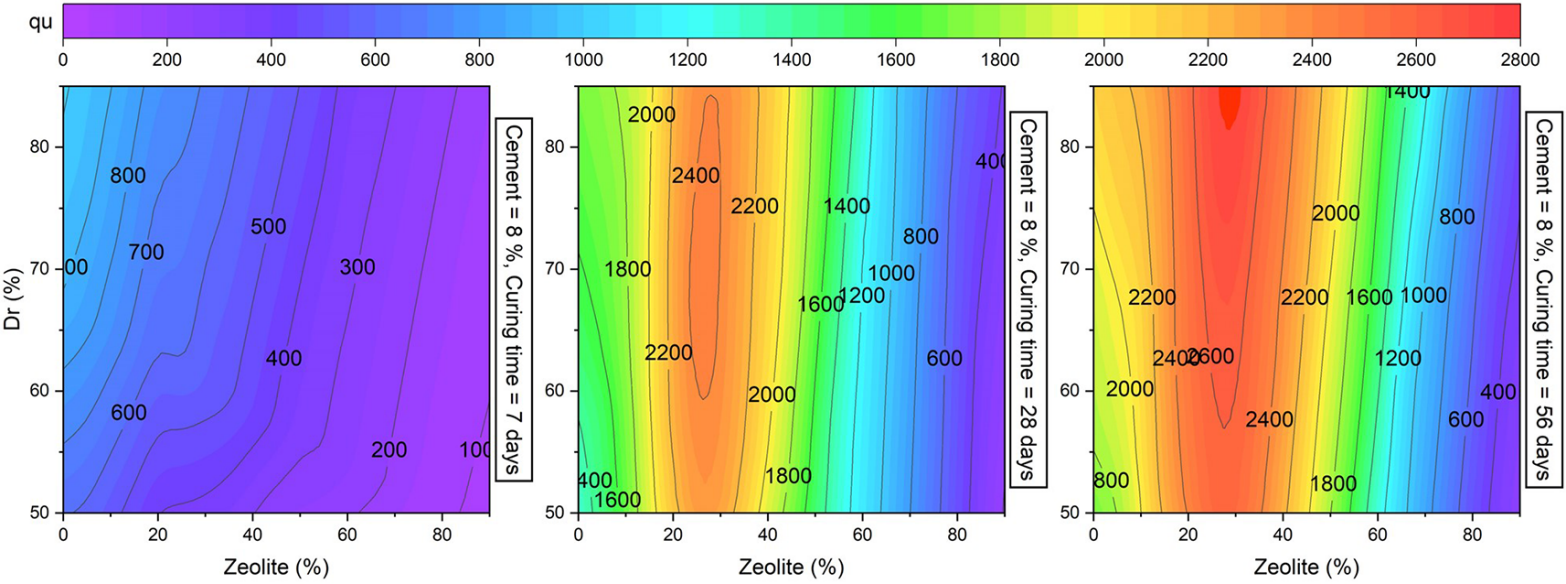
Multivariate contour of UCS versus Zeolite and *Dr.*

**Figure 19.**
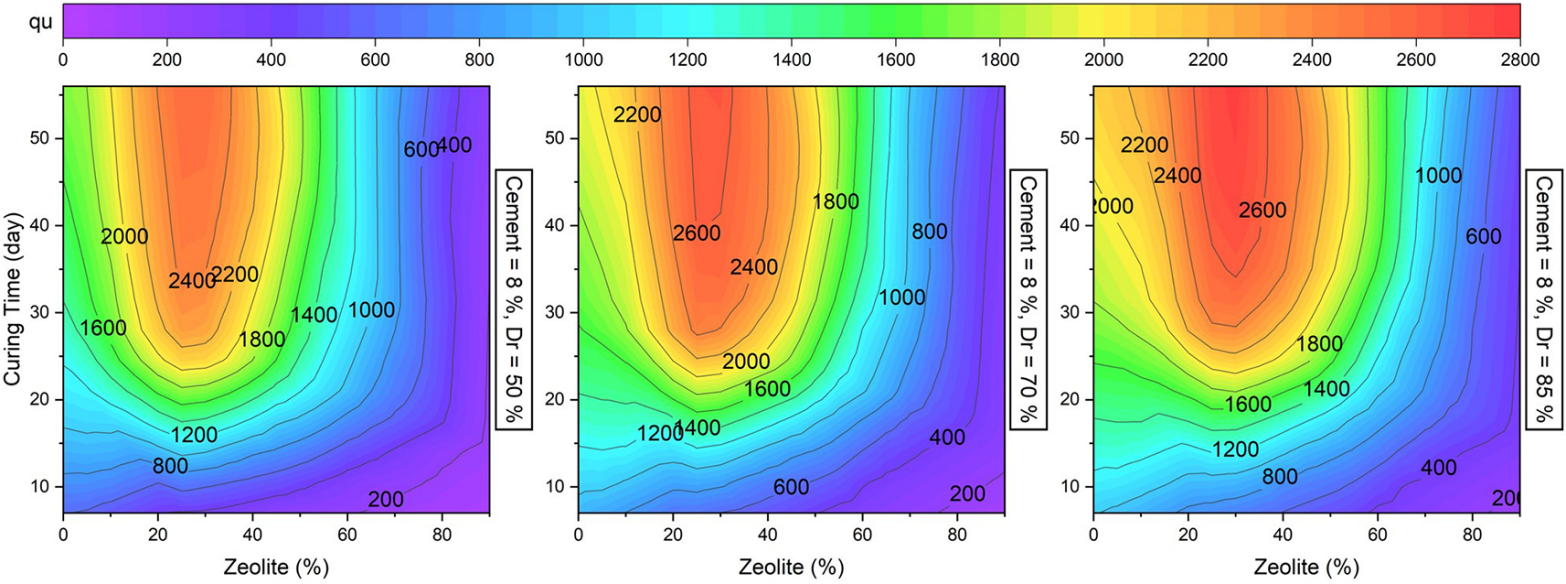
Multivariate contour of UCS versus zeolite and curing time.

To further investigate the sensitivity of *q _u_* to variation in input parameters including *Dr* and zeolite replacement level, Figure 18 presents *q _u_* versus the zeolite content and *Dr* at different values of curing time, at a constant value of cement. It can be observed that increasing the zeolite replacement to 25-30%, can increase the *q _u_* up to 2500 and 2800 kPa for 28 and 56 days of curing time, respectively. In addition, increasing the zeolite replacement by more than 35% decreased the strength of the cemented sand. Figure 17 and Figure 18 also signify that the strength of sand was more sensitive to cement addition and zeolite replacement in comparison with changes in the relative density. Figure 19 illustrates the effect of zeolite replacement and curing time on *q _u_.* It can be concluded that *q _u_* increased by increasing the curing time. Also, this figure shows that increasing the curing time beyond 40 days did not effectively improve the strength of sand. As mentioned earlier, the optimal value of the Zeolite replacement was in range of 20-35% of the cement content.

**Figure 20.**
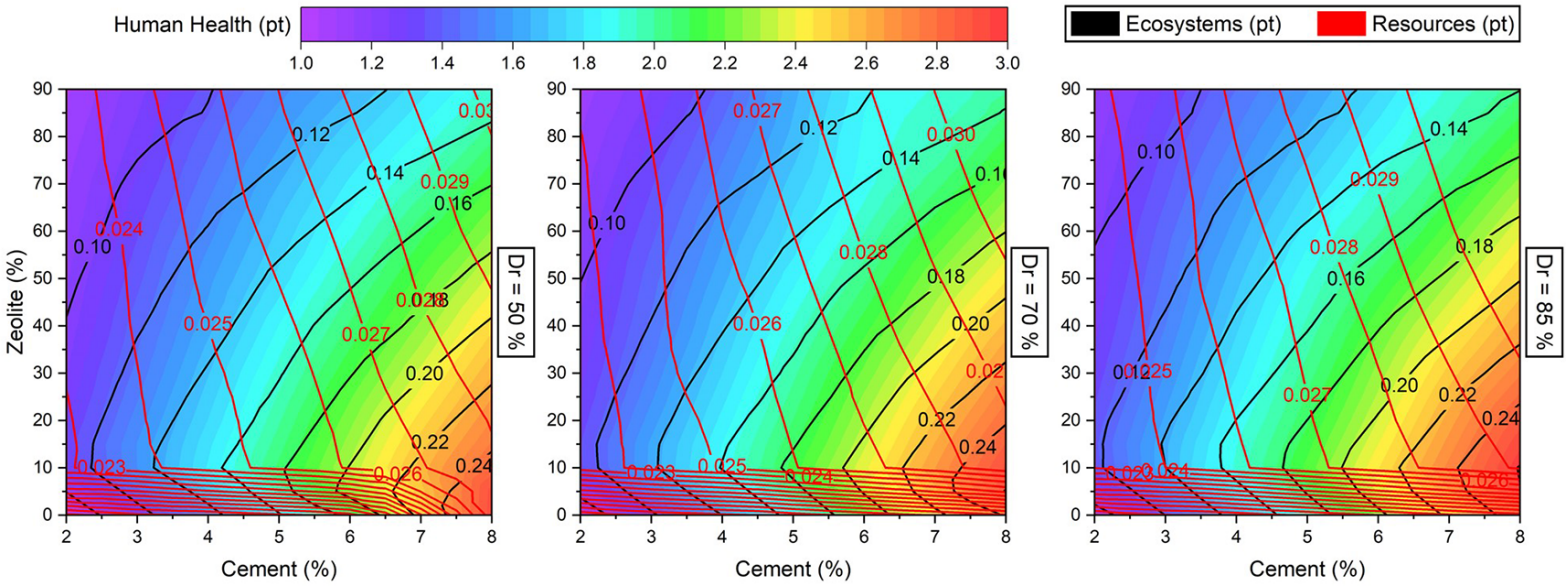
Multivariate contour of LCA values (human health, ecosystems, and resources) versus cement and zeolite.

Figure 20 illustrates contour maps of the LCA values for changes in cement, zeolite partial replacement for cement, and *Dr.* It can be observed that all three LCA values of human health, ecosystem, and resources increased with increasing cement dosage. Furthermore, increasing the zeolite replacement was effective at reducing the human health ecosystem negative effects, but slightly increased the effect of resources. It is worth noting that the LCA values are not very affected by changing the relative density. A key consideration in analyzing the sensitivity of LCA for this sand-cement-zeolite system is not only to consider the effect of these variables on LCA, but also to pay attention to the combined effects on the mechanical strength. In other words, this combined effect can not only be analyzed by these sensitivity graphs. In this respect, multi-objective optimization can help to find a combination of variables to minimize the economic and ecological effects, while maximizing the mechanical strength. Multi-objective optimization also can be organized to find the minimal cost and LCA for a desirable soil strength.

### Optimal design and decision making

**Figure 21.**
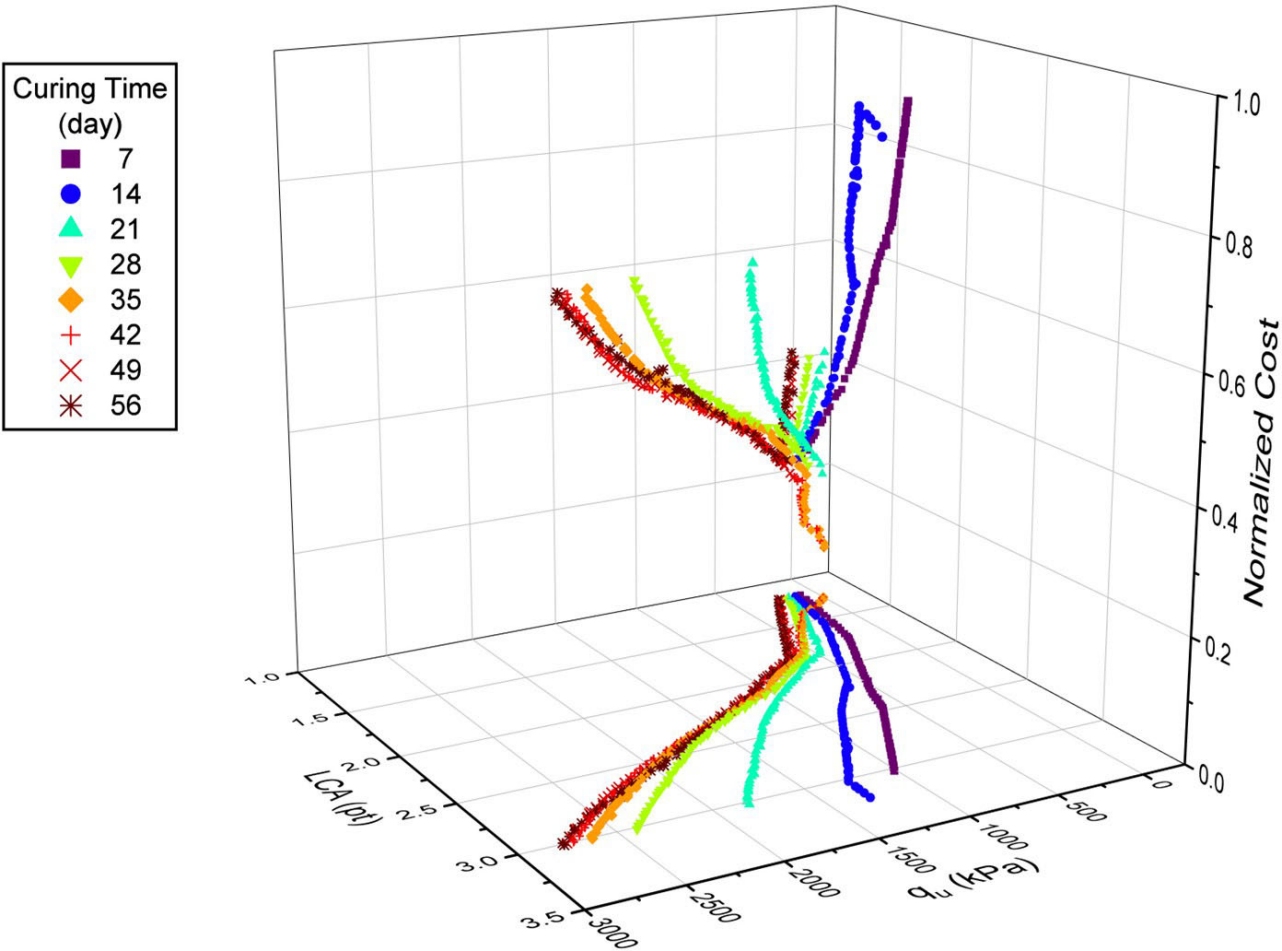
Optimal Pareto front for various curing times.

**Figure 22.**
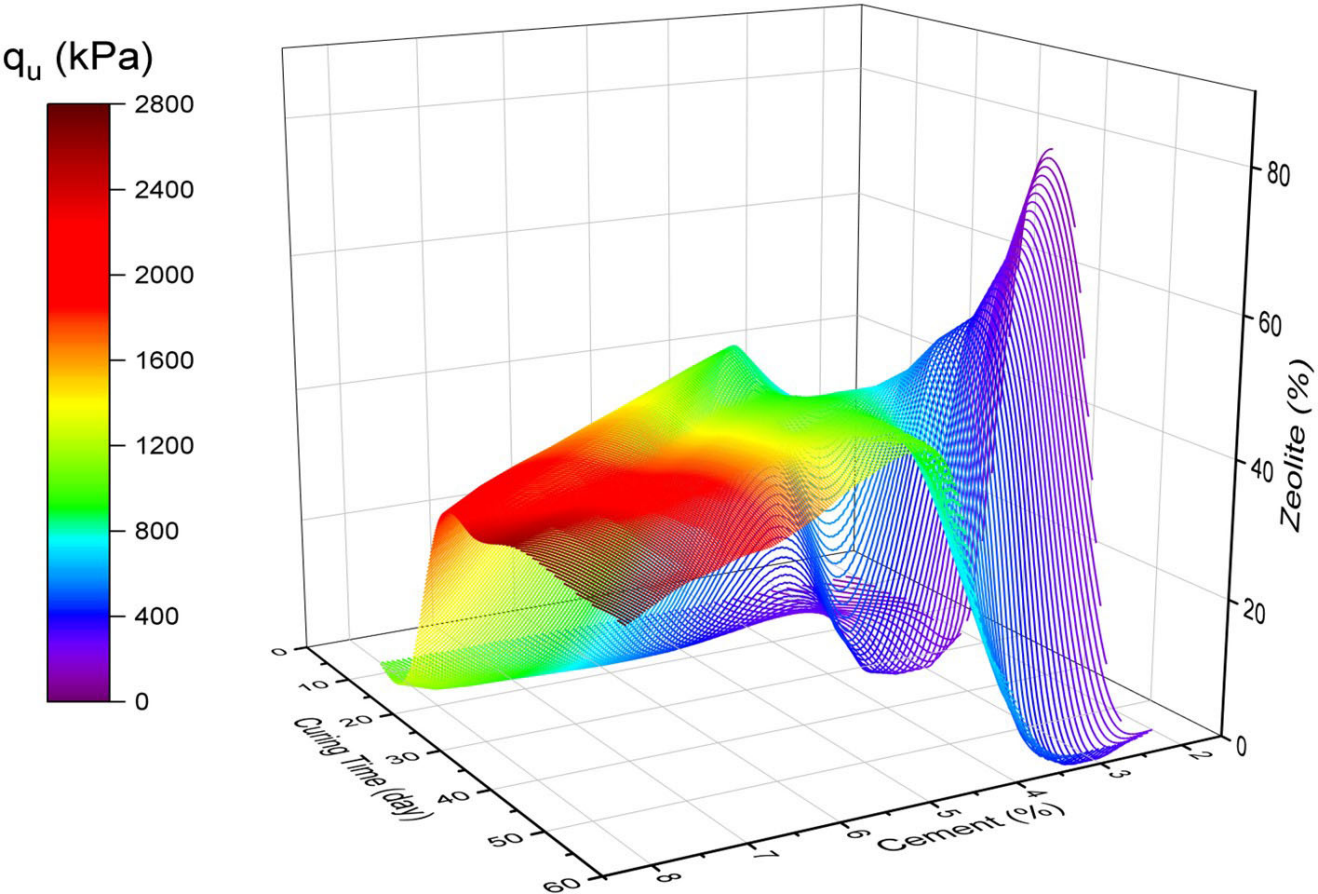
Optimal surface of UCS in the variables space.

The optimization framework based on AGE-MOEA aims to maximize the unconfined compressive strength of improved sand, while simultaneously minimizing the cost and LCA. Various relative density levels were used to investigate the effect of *Dr.* Figure 21 depicts the Pareto front of the optimization problem for 100 optimal points for each *Dr* percentage, as well as the corresponding projection on *q _u_* and LCA plan. It can be observed that optimal cost and LCA values increased for higher optimal values of *q _u_.* Also, for equal optimal values of *q _u_*, the LCA and cost decreased by increasing the curing time up to almost 35-40 days. Considering these observations and sensitivity analysis, the effective curing time was in the range of 30 to 40 days. Optimal values of parameters are used to illustrate the optimal surfaces as shown in Figure 22 to Figure 24. It can be concluded that for the effective curing time, the optimal value of zeolite replacement for cement is around 30-45%, which is in line with previous findings. Also, the trend of cost and LCA is almost the same, which mostly increased by increasing the cement percentage. The parallel plot of the Pareto optimal solutions is depicted in Figure 25. It can be concluded that the optimal range of zeolite (30-45%) not only reduced the cost and LCA, but also narrowed the cost and LCA range while increasing the strength.

**Figure 23.**
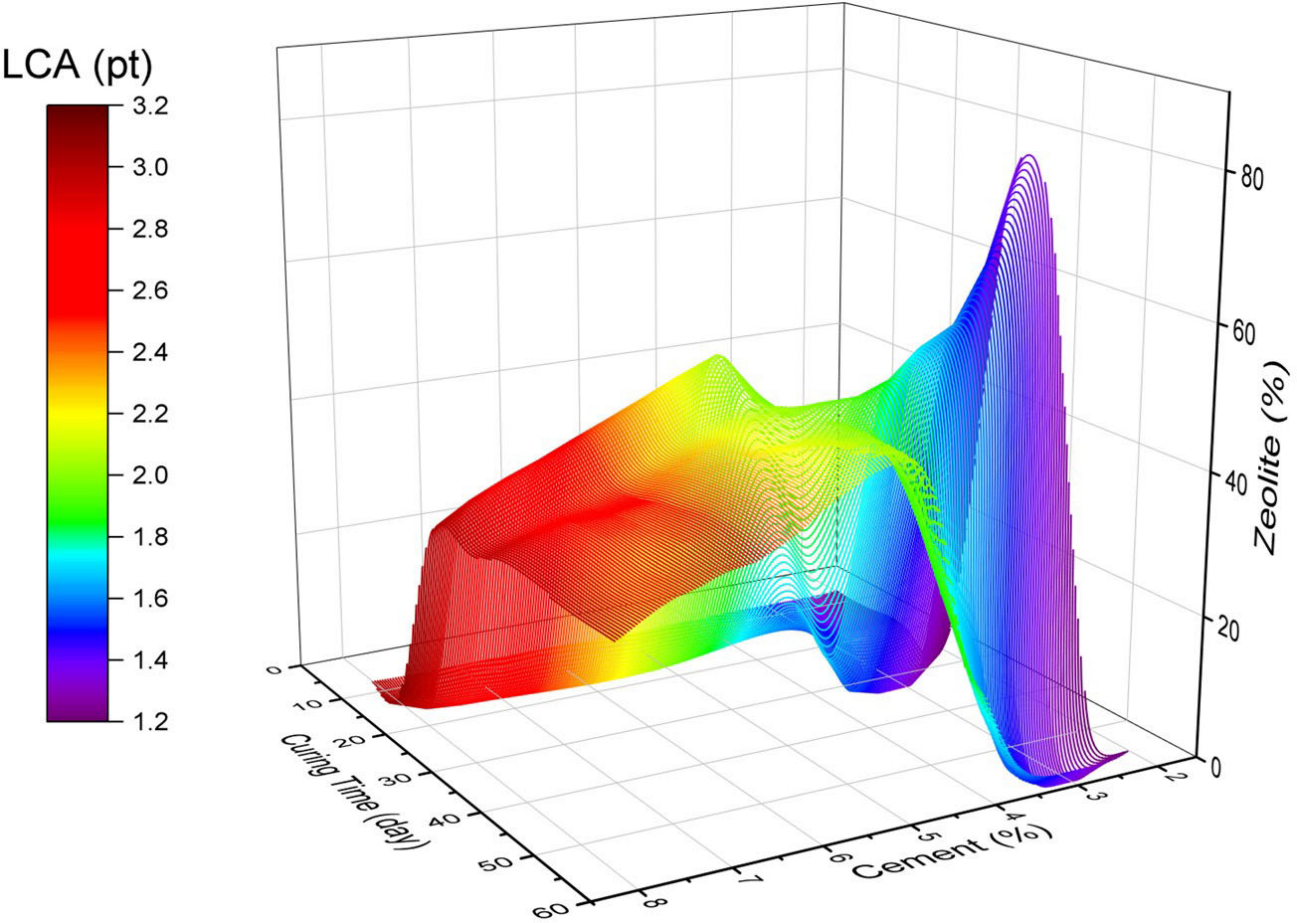
Optimal surface of LCA in the variables space.

**Figure 24.**
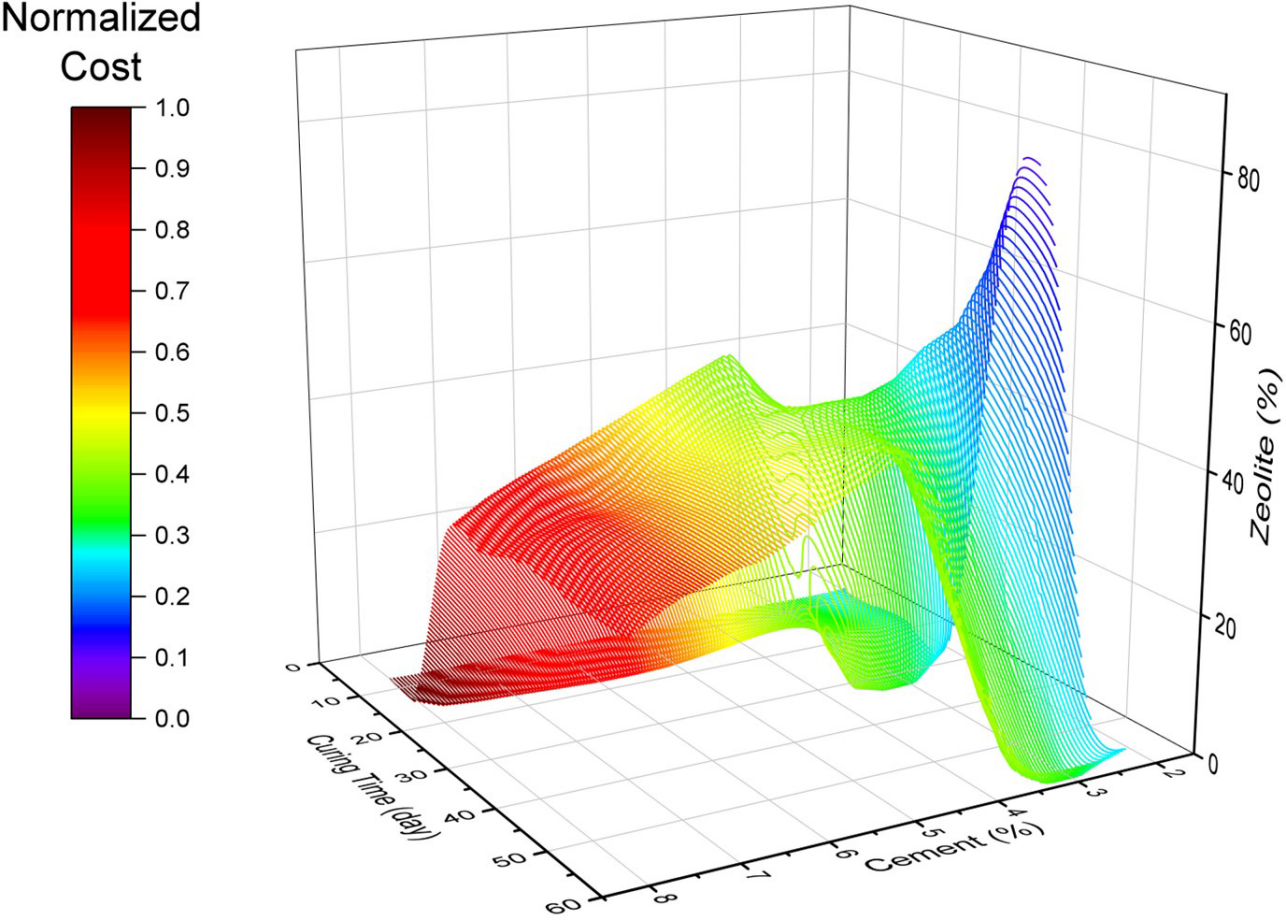
Optimal surface of cost in the variables space.

**Figure 25.**
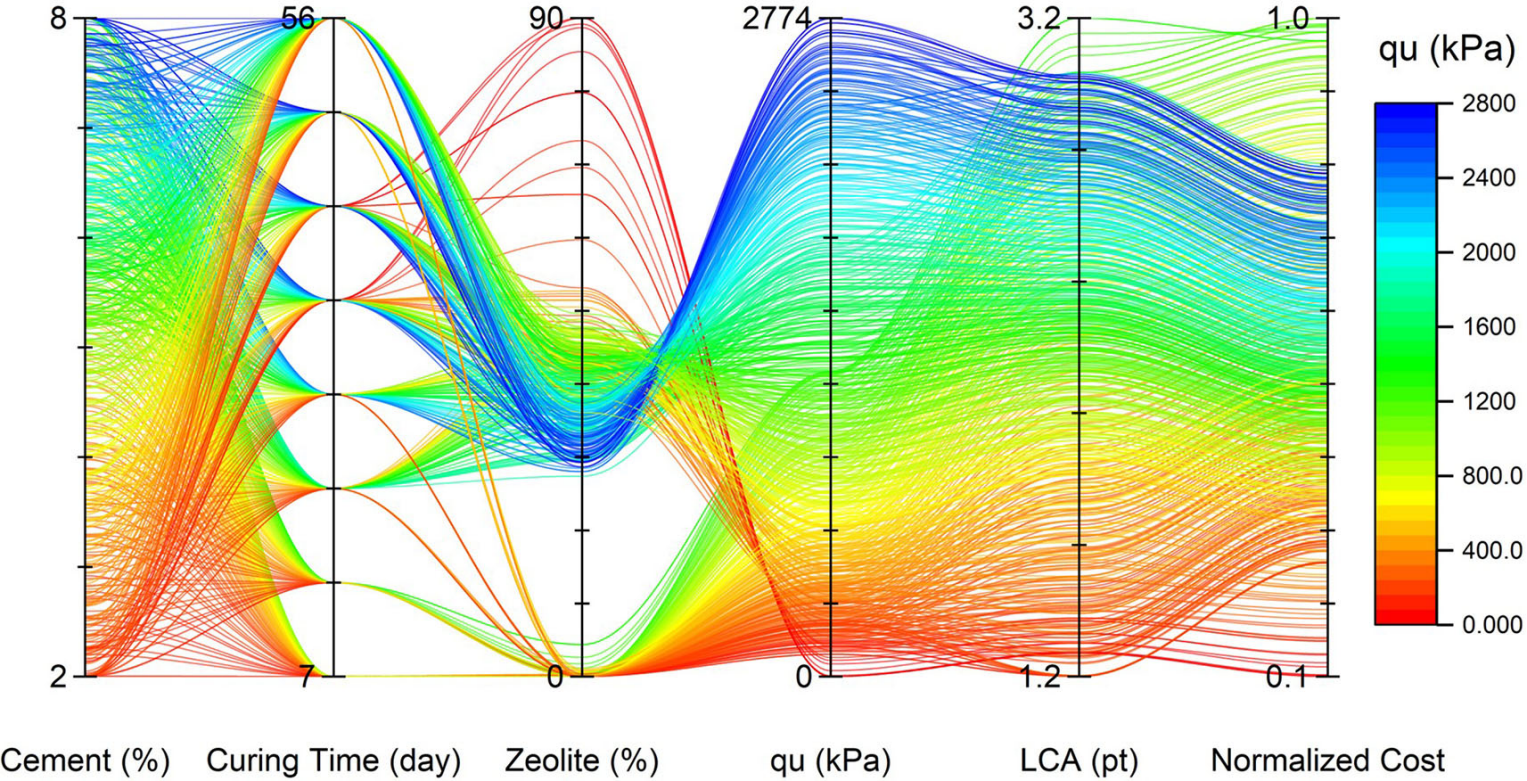
Values of design variables and objective functions for optimal solutions.

To simplify the decision-making process, the optimal values of design variables are reported in Table 5 for desired target *q _u_* at different curing times. Also, the minimum and maximum range of possible optimal solutions for each value of curing time are reported in Table 6. For each level of target *q _u_*, the decision maker can choose the intended combination of design variables by choosing desirable objective functions such as LCA or cost. For example, if the decision maker intends to find a solution for *q _u_* of 1000 kPa with lowest LCA, this can be reached by adding 4.8% cement and 46.6% zeolite.

**Table 5.** Values of design variables and objective functions for different target levels of UCS.

q _u_ level	Curing time	Cement	Zeolite	q _u_	LCA	Normalized cost
200	7	3.5	0.0	201	1.656	0.43
200	14	2.9	0.0	200	1.459	0.35
200	21	2.4	0.0	202	1.298	0.29
200	28	2.0	0.0	200	1.199	0.25
500	7	5.4	0.0	498	2.218	0.67
500	14	4.8	0.0	503	2.043	0.59
500	21	4.0	48.8	505	1.854	0.32
500	28	3.8	0.0	500	1.739	0.47
500	35	3.5	50.5	501	1.741	0.27
500	42	3.2	44.1	497	1.695	0.26
500	49	3.4	0.0	501	1.602	0.41
500	56	3.2	0.1	494	1.567	0.40
1000	7	7.9	0.0	991	2.993	0.98
1000	14	6.8	0.0	1001	2.662	0.85
1000	21	5.3	41.5	1001	2.183	0.46
1000	28	5.1	43.1	1032	2.122	0.44
1000	35	5.0	45.4	991	2.089	0.42
1000	42	4.8	44.7	995	2.033	0.40
1000	49	4.8	46.6	998	2.017	0.40
1000	56	4.8	44.7	999	2.031	0.40
1500	21	6.8	33.7	1488	2.634	0.63
1500	28	5.7	38.0	1485	2.333	0.52
1500	35	5.8	41.1	1497	2.301	0.50
1500	42	5.7	40.4	1498	2.293	0.50
1500	49	5.8	42.8	1501	2.295	0.50
1500	56	5.8	41.5	1518	2.312	0.51
2000	28	7.0	34.4	2010	2.683	0.65
2000	35	6.5	36.3	1991	2.549	0.59
2000	42	6.5	37.7	2008	2.517	0.58
2000	49	6.5	37.5	2000	2.531	0.58
2000	56	6.7	36.7	2027	2.580	0.60
2500	49	7.4	32.6	2500	2.801	0.70
2500	42	7.5	32.5	2509	2.834	0.71
2500	56	7.4	31.7	2490	2.814	0.71
2500	35	7.7	30.7	2511	2.901	0.74

**Table 6.** Values of design variables and objective functions for minimum and maximum possible target levels of UCS.

*q _u_* level	Curing time	Cement	Zeolite	*q _u_*	LCA	Normalized cost
minimum	7	2.0	0.0	94	1.197	0.25
	14	2.0	0.0	128	1.197	0.25
	21	2.0	0.0	172	1.197	0.25
	28	2.0	0.0	199	1.197	0.25
	35	2.0	49.3	239	1.398	0.16
	42	2.0	44.3	255	1.412	0.17
	49	2.2	0.0	265	1.253	0.27
	56	2.2	0.0	271	1.236	0.26
maximum	7	8.0	0.0	1012	3.021	1.00
	14	8.0	4.4	1285	3.185	0.96
	21	8.0	27.4	1817	3.022	0.79
	28	8.0	28.0	2406	3.016	0.79
	35	8.0	28.6	2631	3.009	0.79
	42	8.0	29.2	2723	3.002	0.78
	49	8.0	29.3	2754	3.002	0.78
	56	8.0	28.6	2774	3.009	0.79

## Conclusions

This study presents a promising cement replacement alternative for soil improvement projects. The use of zeolite, a naturally occurring mineral, can significantly reduce the environmental impact of soil strengthening and stabilization, a widespread engineering practice. This study’s experimental and analytical findings shed light on the optimal combination of cement, zeolite, compaction, and curing time to achieve maximum soil strength, lower cost, and better environmental indices. Results indicate that zeolite can partially replace cement in applications involving cemented sand while maintaining comparable or even superior mechanical strength performance.

The proposed BPNN model was found to be highly accurate in predicting the ultimate strength of cemented sand, and the model’s optimal architecture was identified through the tuning process. Moreover, the application of the Adaptive Geometry Estimation based on the Multi-Objective Evolutionary Algorithm (AGE-MOEA) enabled the identification of the optimal values of cement, zeolite, density, and curing time by simultaneously minimizing environmental impact and cost while optimizing strength. This study’s findings provide a basis for future research and development of sustainable soil improvement practices in the construction industry. The BPNN model developed in this study accurately predicts the ultimate strength of cemented sand. The A-2 architecture of BPNN with 200 and 50 units is superior to other architectures in terms of statistical metric values and stability for predicting the ultimate strength of the studied sand. Increasing the neural network’s depth and size does not improve the accuracy of the BPNN model for this particular problem, as shown by the results.

Increasing the zeolite replacement to 25-30% can increase the ultimate strength of cemented sand, but exceeding this limit can cause the strength to decrease. In addition, the relative density of sand is less sensitive to cement addition and zeolite replacement than its strength.

The LCA analysis demonstrates that increasing the cement dosage increases the LCA values, whereas increasing the zeolite replacement decreases adverse effects on human health and ecosystems. From an LCA standpoint, using zeolite as partial cement replacement in soil improvement projects is a promising alternative.

The framework for optimization based on AGE-MOEA demonstrates that the optimal range of zeolite replacement for cement is between 30 and 45%, reducing cost and LCA, while increasing mechanical strength. By minimizing the environmental impact and cost, while maximizing strength, the optimal range of zeolite not only reduces cost and LCA, but also reduces the range of these variables and increases the strength of cemented sand.

The optimal values of cement and zeolite for various curing times and target *q _u_* levels are provided, which can be used to facilitate decision-making and optimize the performance of system design.

Zeolite utilization is a promising alternative for partial replacement of cement in soil improvement projects from an LCA perspective.

## Data Availability

Harvard Dataverse. Optimized Mixtures and LCA Metrics: Cement-Zeolite Improved Sand Data, https://doi.org/10.7910/DVN/KNOSM4, Harvard Dataverse, V1. This project contains the following underlying data
•

Zeolite_Data_LCA.xlsx (This file contains the composition, performance metrics, and environmental impact assessments of cement-zeolite improved sand mixtures. It includes data on material compositions, Life Cycle Assessment (LCA) results, and unconfined compressive strength of the mixtures. More details on the data headers and their implications are available in the linked data repository.) Data are available under the terms of the Creative Commons Zero “No rights reserved” data waiver (CC0 1.0 Public domain dedication).

## Software availability

The computational tasks in this study were achieved through the utilization of the open-source libraries TensorFlow (licensed under Apache License 2.0) and PyMOO (licensed under Apache License 2.0) ^58^ in Python. In addition, the SimaPro software was used for a detailed life cycle assessment.

**Source code available from:** https://zenodo.org/records/10079343

**Archived source code at time of publication:** https://zenodo.org/doi/10.5281/zenodo.10079342 ^59^

**License:** Creative Commons Attribution 4.0 International
