# Supplementary material for: Multi-objective optimization of sustainable cement-zeolite improved sand based on life cycle assessment and artificial intelligence
Source: F1000Res. 2024 Apr 10;13:257. [Version 1] doi: 10.12688/f1000research.148275.1 (PMC12375181; doi:10.12688/f1000research.148275.1)
Supplement: Peer review history

# Reviewer report: Reviewer response for version 1

**DOI:** 10.5256/f1000research.162564.r401790

**Published:** 2025-08-23

**Authors:** Asma Thamir Ibraheem

**Affiliations:** 1 Al-Nahrain University, Baghdad, Iraq

1. Using BPNN, evolutionary algorithms, and life cycle evaluation, the work presents a valuable and integrated strategy to optimise cement-zeolite enhanced sand. A distinct advantage is the integration of mechanical, economic, and ecological goals.
2. There are different types of natural zeolite. Which type is used in this research? Show the mechanical and physical properties.
3. Show the optimum percentage of zeolite replacement. Experimentally, the rates of 50, 70, and 90% of zeolite gave counterproductive results.
4. The results are comprehensive but may be condensed to emphasize the most critical facts and engineering-related implications.
5. The results are backed up by carefully selected figures and tables.
6. A great way to help with reproducibility is to make data and code openly available.
7. Several measures show that the prediction model is performing well statistically.
8. While most of the manuscript is well-written, the sections devoted to results and discussion are lengthy.

Is the work clearly and accurately presented and does it cite the current literature?

Yes

If applicable, is the statistical analysis and its interpretation appropriate?

Yes

Are all the source data underlying the results available to ensure full reproducibility?

Yes

Is the study design appropriate and is the work technically sound?

Yes

Are the conclusions drawn adequately supported by the results?

Partly

Are sufficient details of methods and analysis provided to allow replication by others?

Yes

Reviewer Expertise:

Civil Engineering, Transportation and Airports Engineering, Concrete Pavement

I confirm that I have read this submission and believe that I have an appropriate level of expertise to confirm that it is of an acceptable scientific standard, however I have significant reservations, as outlined above.

# Reviewer report: Reviewer response for version 1

**DOI:** 10.5256/f1000research.162564.r401795

**Published:** 2025-08-22

**Authors:** Erol Yilmaz

**Affiliations:** 1 Recep Tayyip Erdogan University, Rize, Turkey

The article, as currently submitted, demonstrates a high level of scholarly quality and can be accepted for publication in its present form. The topic is timely and relevant, and the authors have presented their findings in a clear, well-organized, and scientifically sound manner. The methodology is appropriate, and the analysis is thorough, with meaningful interpretations of the results.

 The literature review is comprehensive and effectively sets the context for the study. Figures and tables are generally well-prepared and contribute to the overall clarity of the manuscript. The discussion reflects a solid understanding of the subject and highlights the practical implications of the work.

 While the paper does not require further revision, the authors may consider enhancing the visibility of their key findings in the conclusion section to further emphasize the novelty and potential applications of their research.

 Overall, this is a commendable piece of work that makes a valuable contribution to the field. Congratulations to the authors.

Is the work clearly and accurately presented and does it cite the current literature?

Yes

If applicable, is the statistical analysis and its interpretation appropriate?

Yes

Are all the source data underlying the results available to ensure full reproducibility?

Yes

Is the study design appropriate and is the work technically sound?

Yes

Are the conclusions drawn adequately supported by the results?

Yes

Are sufficient details of methods and analysis provided to allow replication by others?

Yes

Reviewer Expertise:

I am working as full university at Recep Tayyip Erdoğan University in Rize, Türkiye, focusing on construction and building materials

I confirm that I have read this submission and believe that I have an appropriate level of expertise to confirm that it is of an acceptable scientific standard.

# Reviewer report: Reviewer response for version 1

**DOI:** 10.5256/f1000research.162564.r382175

**Published:** 2025-05-28

**Authors:** Mohammad Javad Moradi

**Affiliations:** 1 Department of Civil and Environmental Engineering, Carleton University, Ottawa, Canada

The study offers a valuable and well-integrated approach to optimizing cement-zeolite improved sand using BPNN, evolutionary algorithms, and life cycle assessment. The combination of mechanical, economic, and environmental objectives is a clear strength.

     1.    The conclusions are thorough but could be shortened to highlight only the most impactful findings and practical takeaways for engineering applications.

     2.    The manuscript is mostly well-written, but the results and discussion sections are quite dense. Consider tightening some sentences for better readability and clarity.

     3.    Figures and tables are well chosen and clearly support the findings.

     4.    Open access to data and code is a valuable contribution to reproducibility.

     5.    The predictive model is strong, with good statistical performance across multiple metrics.

     6.    Please add 1–2 sentences justifying the use of BPNN instead of other ML models (e.g., Random Forest, SVM) and clarify whether comparative testing was performed.

      7. Please cite following studies related to submitted manuscript:

 a) Predicting the compressive strength of concrete containing metakaolin with different properties using ANN

 b) Application of artificial intelligence in predicting the residual mechanical properties of fiber reinforced concrete (FRC) after high temperatures

 Recommendation: After addressing these minor revisions, the manuscript is suitable for indexing.

Is the work clearly and accurately presented and does it cite the current literature?

Partly

If applicable, is the statistical analysis and its interpretation appropriate?

Yes

Are all the source data underlying the results available to ensure full reproducibility?

Yes

Is the study design appropriate and is the work technically sound?

Yes

Are the conclusions drawn adequately supported by the results?

Yes

Are sufficient details of methods and analysis provided to allow replication by others?

Partly

Reviewer Expertise:

Machine learning

I confirm that I have read this submission and believe that I have an appropriate level of expertise to confirm that it is of an acceptable scientific standard, however I have significant reservations, as outlined above.

## References

[1] : Predicting the Compressive Strength of Concrete Containing Binary Supplementary Cementitious Material Using Machine Learning Approach. *Materials (Basel)* .2022;15(15) : 10.3390/ma15155336 10.3390/ma15155336 35955269 PMC9369809
